# Remodeling mechanisms and intervention strategies of the oral mucosal immune barrier

**DOI:** 10.3389/fimmu.2026.1815513

**Published:** 2026-05-28

**Authors:** Zun Wang, Yaohua Guo, Yaguang Fan, Xuebing Li, Wang Shen

**Affiliations:** 1 Sichuan University, West China School of Stomatology, Chengdu, China; 2 West China Hospital of Sichuan University, Chengdu, China; 3 Tianjin Medical University General Hospital, Tianjin Lung Cancer Institute, Department of Lung Cancer Surgery, Tianjin Key Laboratory of Lung Cancer Metastasis and Tumor Microenvironment, Tianjin, China

**Keywords:** barrier function, epithelial barrier, immune remodeling, intervention strategies, microbiota, oral mucosal immunity

## Abstract

The oral mucosa serves as the primary line of defense against exogenous pathogens and antigens, and its barrier function is critical for both local oral health and systemic well-being. The oral mucosal barrier forms a multilevel defense system consisting of physical, chemical, microbial, and cellular barriers, and its dynamic remodeling relies on an intricate network among epithelial cells, immune cells and the microbiota. Epithelial cells act as immune sentinels to coordinate immune responses, while innate and adaptive immune cells mediate immediate defense, precise regulation and immune memory respectively. The oral microbiota also consolidates the barrier function by resisting colonization of pathogens, and through metabolic activities and other pathways. This finely balanced network can be perturbed by local factors such as infection, autoimmune oral diseases and oral cancer, as well as systemic factors including diabetes and tumor chemoradiotherapy, leading to barrier dysfunction. A dysfunctional oral mucosa can exacerbate local lesions, and induce or worsen systemic metabolic and inflammatory diseases through the oral-systemic axis. In recent years, the application of cutting-edge technologies such as single-cell sequencing and spatial transcriptomics has deepened our understanding of the molecular and cellular mechanisms underlying oral barrier remodeling, and spurred the development of targeted and diversified intervention approaches, including microbiota-targeted regulation, immune signaling pathway modulation, soluble biological scaffolds, and nanocarrier-based drug delivery. This review elaborates on the composition and remodeling processes of the oral mucosal immune barrier, the consequences of barrier dysfunction, and the novel therapeutic strategies for its restoration and regulation. It also highlights the current limitations in research, such as the lack of integrated mechanistic studies and standardized protocols, which warrants integration of basic research and clinical practice. The aim of this review is to guide the development of novel therapies for oral mucosal barrier-related diseases and provide a scientific basis for the integrated management of oral and systemic health.

## 1 Introduction

The oral mucosa is a complex and dynamic microenvironment comprising of physical, chemical, microbial, and cellular barriers (1). In addition to defending against pathogen invasion and maintaining tissue homeostasis, the oral mucosal barrier also plays a pivotal role in inducing immune tolerance (2). As the largest mucosal surface exposed to the external environment, the oral mucosa is constantly subjected to physical and chemical stimuli, and the risk of invasion by a vast array of microorganisms, making its barrier function indispensable for preserving oral and even systemic health (3). In this review, we have defined barrier remodeling as the reorganization of the epithelial architecture, junctional composition, local immune microenvironment, microbiota-host interactions, and saliva- and mucos-associated chemical defenses in response to physiological turnover, environmental exposure, and pathological challenges, which ultimately maintain or restore barrier homeostasis (1). In contrast, barrier disruption refers to an acute or directly induced structural breach of the epithelial or mucus-salivary barrier, whereas barrier dysfunction is defined as the sustained loss of barrier competence, characterized by increased permeability, defective antimicrobial defense, aberrant immune tone, or delayed healing (1, 2).

Oral mucosal barrier remodeling involves four interrelated processes: structural remodeling of the epithelium and intercellular junctions, immunological remodeling involving resident and infiltrating immune cells and their cytokine networks, ecological remodeling of the commensal-pathogen balance and microbial metabolites, and functional remodeling reflected by altered permeability, antimicrobial capacity, immune tolerance, and tissue-repair output. The net result of this integrated process determines whether the barrier returns to homeostasis or progresses toward chronic inflammation, defective healing and persistent barrier dysfunction.

Physiological remodeling refers to constitutive epithelial turnover, junctional renewal, saliva-mucus replenishment, and tonic immune education by commensal microorganisms that preserve barrier homeostasis. On the other hand, adaptive remodeling is the reversible and protective response to transient challenge, characterized by controlled epithelial restitution, temporary immune activation, antimicrobial reinforcement, and ecological recalibration that favor recovery. In contrast, pathological remodeling describes a disease-associated reprogramming process in which persistent inflammatory signaling, aberrant epithelial differentiation, stromal activation, and microbial dysbiosis reshape the barrier niche. When these alterations become self-perpetuating and no longer restore competence, they culminate in maladaptive barrier disruption, which is marked by sustained permeability, defective healing, immune miseducation, and chronic inflammatory niche formation. Thus, oral barrier remodeling should not be interpreted solely as a structural epithelial event, but as a coordinated process involving epithelial architecture, immune-cell state transitions, microbial ecological shifts, and the progressive establishment or resolution of inflammatory tissue niches.

Persistent or repetitive barrier disruption may drive maladaptive remodeling and ultimately culminate in barrier dysfunction, which in turn triggers or exacerbates the development of both oral and systemic diseases (1). For instance, in erosive and ulcerative oral mucosal diseases such as oral lichen planus, recurrent aphthous ulcer and pemphigus, abnormal T cell activation leads to direct cytotoxicity and cytokine-mediated damage, which impairs epithelial stem cell function and damages the basement membrane, ultimately causing persistent barrier failure (4). Similarly, chronic diseases like oral submucous fibrosis are closely linked to epithelial barrier injury, with mitochondrial dysfunction and tight junction disruption being key pathogenic events (5).

The oral barrier is not merely an extension of the intestinal mucosa; it is organized across keratinized and non-keratinized subsites, and is continuously bathed by saliva. Furthermore, it also interfaces with tooth- and implant-associated hard surfaces that support biofilms, and experiences repeated mechanical challenge from mastication and speech. Accordingly, barrier renewal, antimicrobial chemistry, and immune set-points differ across gingival, buccal, palatal, and tongue mucosa. Accumulating evidence indicates that oral keratinocytes not only form the structural building blocks of the physical barrier, but also regulate immune responses by recognizing pathogenic microorganisms via pattern recognition receptors (PRRs), producing antimicrobial peptides (AMPs) and cytokines, and presenting antigens (6). In fact, keratinocytes are the major effector cells of the innate immune response in the oral mucosa, and their functions are strongly influenced by the unique oral microenvironment and resident microbiota (3). Furthermore, the oral and gastrointestinal mucosal immune niches differ in terms of anatomical features, cell communication, antigen processing, and signal transduction, which underpins their independent yet interdependent functions under homeostatic and stress conditions (2). Accordingly, we have focused on the immune mechanisms specific to oral tissues, whereas findings from other mucosal systems have been discussed only as supportive analogies in case of limited direct evidence on the oral milieu. Following acute barrier disruption, the inflammatory cytokine network is activated to coordinate wound healing, a process that can be “hijacked” by malignant transformation, leading to oral carcinogenesis (7). Meanwhile, studies have linked persistent barrier dysfunction to a wide range of systemic diseases, including cardiovascular, neurodegenerative and metabolic disorders, although the specific molecular networks underlying this cross-organ communication remain poorly understood (1).

In recent years, advances in single-cell sequencing, spatial transcriptomics, organoid culture and multi-omics integrative analysis have deepened our understanding of the molecular and cellular mechanisms of oral mucosal barrier remodeling. These approaches have shifted the study of oral mucosal immunity from bulk tissue description to cell- and niche-resolved analysis. A foundational single-cell atlas of human oral mucosa delineated discrete epithelial, stromal, myeloid and lymphoid compartments, and linked stromal hyper-responsiveness with neutrophil recruitment in inflammatory lesions, thereby establishing stromal-immune coupling as a central organizing principle of oral barrier immunity (8). More recently, spatial transcriptomic mapping of anatomically distinct oral sites demonstrated that the tongue, cheek and palate harbor reproducible site-specific epithelial-stromal programs and fibroblast growth factor (FGF)-centered niche cues, underscoring that oral barrier remodeling is strongly shaped by local anatomical context rather than representing a uniform mucosal response (9).

In parallel, disease-oriented single-cell and multi-omics studies have begun to define pathogenic cell states with much greater precision. In oral submucous fibrosis, single-cell analysis identified MHC-II^high^ epithelial populations and a proinflammatory/profibrotic epithelial subset that communicates with T cells through MIF-CD74/CXCR4 signaling (10). Furthermore, single cell RNA sequencing (scRNA-seq) of oral leukoplakia lesions revealed an IDO1+ macrophage population associated with an immunosuppressive microenvironment during malignant transformation (11). Likewise, scRNA-seq and immune profiling of oral lichen planus tissues revealed tissue-resident activated memory CD8+ T cells, clonal lymphocyte expansion and fibroblast-associated immune recruitment, and a later single-cell study identified functionally exhausted CD8+ T cell states (12, 13). Visium HD spatial transcriptomics and other single-cell/spatial multi-omics approaches have shown that smoking or chronic periodontal inflammation perturbs epithelial barrier programs, fibroblast-epithelial communication and endothelial-macrophage signaling in gingival disease, and have established epithelial JAG1 and endothelial CXCL12-associated circuits as potentially actionable nodes (14, 15).

Single-cell sequencing can identify rare epithelial subpopulations that are obscured in bulk tissue, such as the stress-responsive, MHC-II^high^, proinflammatory/profibrotic, or differentiation-skewed epithelial states, which can help elucidate which epithelial compartments actively initiate barrier failure (8, 10, 11). Spatial transcriptomics can delineate the regions where these states emerge, and how they interface with fibroblasts, endothelial cells, myeloid cells, and lymphocytes, making it possible to reconstruct immune-spatial interaction circuits such as stromal-neutrophil coupling, fibroblast-mediated lymphocyte recruitment, and endothelial-epithelial signaling gradients across anatomically distinct oral sites (9, 14, 15). When integrated with multi-omics readouts such as immune-repertoire information, pathway activity, and cell-cell communication analysis, these approaches can also map barrier disruption gradients – from relatively intact epithelium to ulcerative, fibrotic, or dysplastic niches – and facilitate biomarker and target discovery by linking discrete cell states and ligand-receptor programs to clinically relevant phenotypes like malignant transformation, therapy-related mucosal injury, and failure of barrier restitution (10–15).

The intervention strategies for barrier dysfunction have also rapidly evolved from traditional anti-inflammatory and antibacterial approaches to precision, microecological and immunomodulatory therapies. Natural products have shown great potential in the treatment of mucosa-associated diseases due to their multi-target and multi-system regulation, and diverse mechanisms of action, such as immunomodulatory and anti-inflammatory effects, restoration of tight junction proteins, and regulation of the gut microbiota (16). In animal models of intestinal inflammation, supplementation with human LYPD8 protein, melatonin, misoprostol or farnesoid X receptor agonists has been proven to alleviate mucosal barrier injury and inflammation through distinct mechanisms (17–20). Nanomaterials hold enormous potential for drug delivery and modulation of intestinal mucosal barrier function in the treatment of intestinal diseases by virtue of their unique physicochemical properties (21). For example, mucoadhesive nanoparticles self-assembled from chitooligosaccharide and glycyrrhizic acid have demonstrated excellent targeted delivery, anti-inflammatory and barrier-restorative effects in inflammatory bowel disease models (22). Targeted interventions against key pathological processes have also been reported, such as the use of nanocarriers that specifically act on goblet cell-associated antigen pathways to maintain colonic mucosal integrity (23), modulation of microRNAs (miRNAs) involved in ferroptosis pathways for inflammatory bowel disease treatment (24), and holistic approaches targeting the oral-gut-brain axis to preserve systemic homeostasis (25, 26). This review summarizes the latest progress in this field, and systematically elaborates on the remodeling of the oral mucosal immune defense barrier to offer new perspectives for intervention strategies.

## 2 Multilevel barrier structure and function of the oral mucosa

### 2.1 Composition and dynamic characteristics of physical and chemical barriers

**Figure 1:**
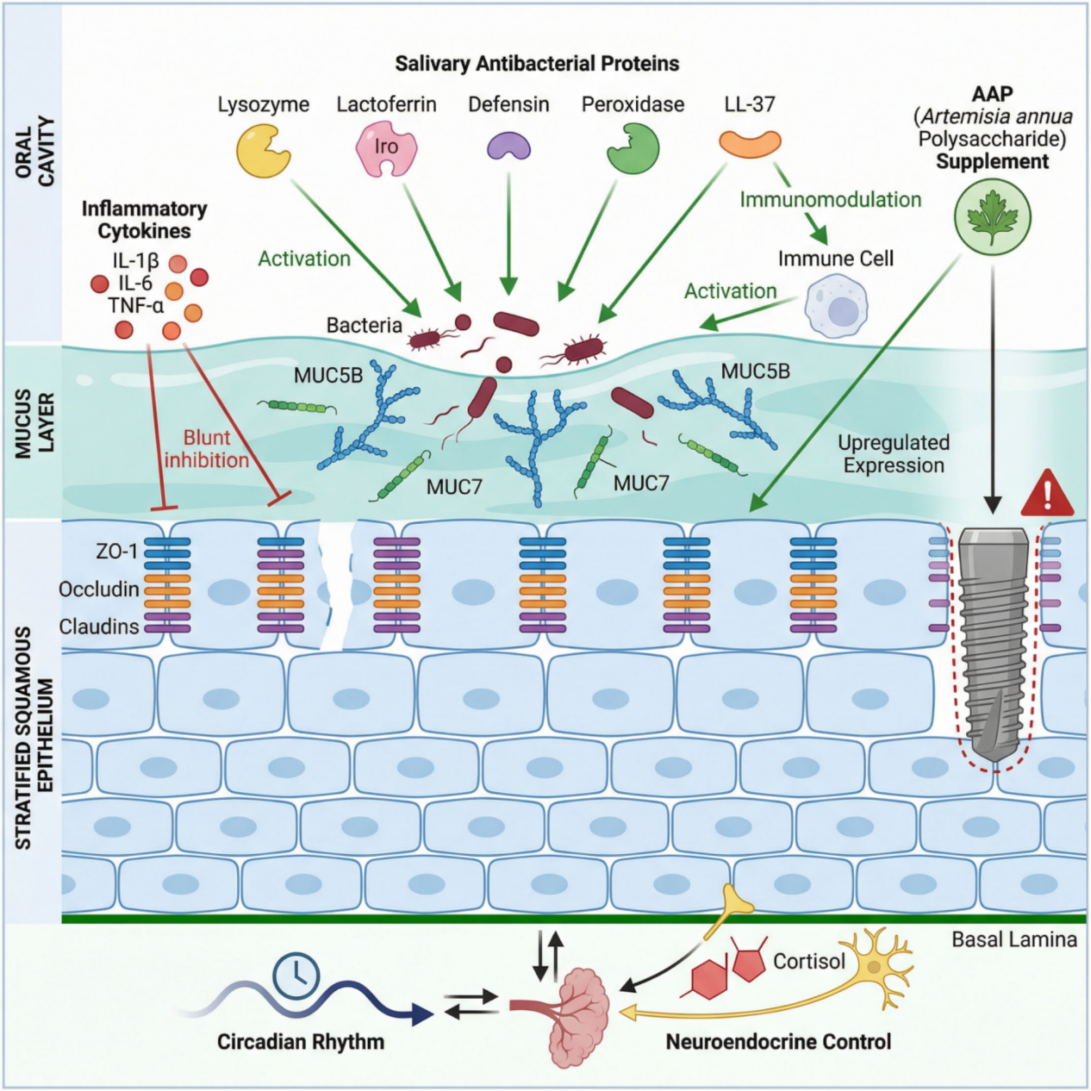
Multilayer structure of the oral mucosal immune barrier, the immune regulatory network, and the intervention mechanism of AAP supplementation.

The oral mucosa constitutes the first line of defense against exogenous invasion. The physical barrier is mainly composed of stratified squamous epithelium, in which epithelial cells form an intact physical barrier via tight junctions, adherens junctions, desmosomes and other intercellular connections. The keratinized or non-keratinized cell layers on its surface undergo constant shedding and renewal, forming a dynamic primary defense barrier (27) (**Figure 1**). This dynamic turnover is crucial for barrier function, particularly in the transmucosal region of dental implants. Compared to natural periodontal tissues, the oral mucosa surrounding implants exhibits lower adhesion to the implant surface, rendering its physical barrier more vulnerable to destruction by oral pathogenic bacteria (27). In addition to structural connections, epithelial cells secrete a mucus layer rich in mucins MUC5B and MUC7, which form a hydrated gel that effectively traps microorganisms, thereby limiting their colonization (28). In addition, the saliva is rich in AMPs and proteins such as lysozyme, lactoferrin, defensins and peroxidase, and therefore functions as a robust chemical barrier (29). For example, the LL-37 peptide, the active form of the human cathelicidin antimicrobial peptide, can directly insert into microbial surface structures to exert antibacterial effects and act as an agonist for various cell membrane receptors to mediate immunomodulation, making it a key molecule for maintaining oral homeostasis (29).

These physical and chemical barriers are not static but finely regulated. The turnover rate of epithelial cells, the expression of intercellular junction proteins (including occludin and claudins), and the flow rate and composition of saliva all exhibit distinct circadian rhythmicity and stress responsiveness under the influence of the neuroendocrine system and local secretory microenvironment (28). There is direct evidence indicating that oral barrier stability is shaped by site-specific epithelial adhesion and junctional regulation: peri-implant mucosa shows weaker attachment and greater vulnerability to bacterial challenge compared to natural periodontal tissue (27), whereas vitamin D3 enhances claudin-4 expression and transepithelial electrical resistance in gingival epithelial cells, supporting a measurable barrier-tightening effect (30). Salivary composition is likewise integral to oral specificity, as changes in mucins, defensins and other antimicrobial proteins alter lubrication, microbial trapping and epithelial protection within the oral cavity (28, 29). Furthermore, under stress or infectious conditions, elevated levels of inflammatory cytokines such as interleukin (IL)-1β, IL-6 and tumor necrosis factor (TNF)-α can impair barrier integrity and modulate the expression of tight junction proteins (31). Thus, oral mucosal barrier function is a highly dynamic and plastic system, and understanding its composition and regulation is crucial for designing strategies to prevent and treat oral mucosal diseases such as oral ulceration, periodontitis and dental implant infection (27, 32).

### 2.2 Microbial barrier: homeostasis and defensive effects of the oral microbiota

**Figure 2:**
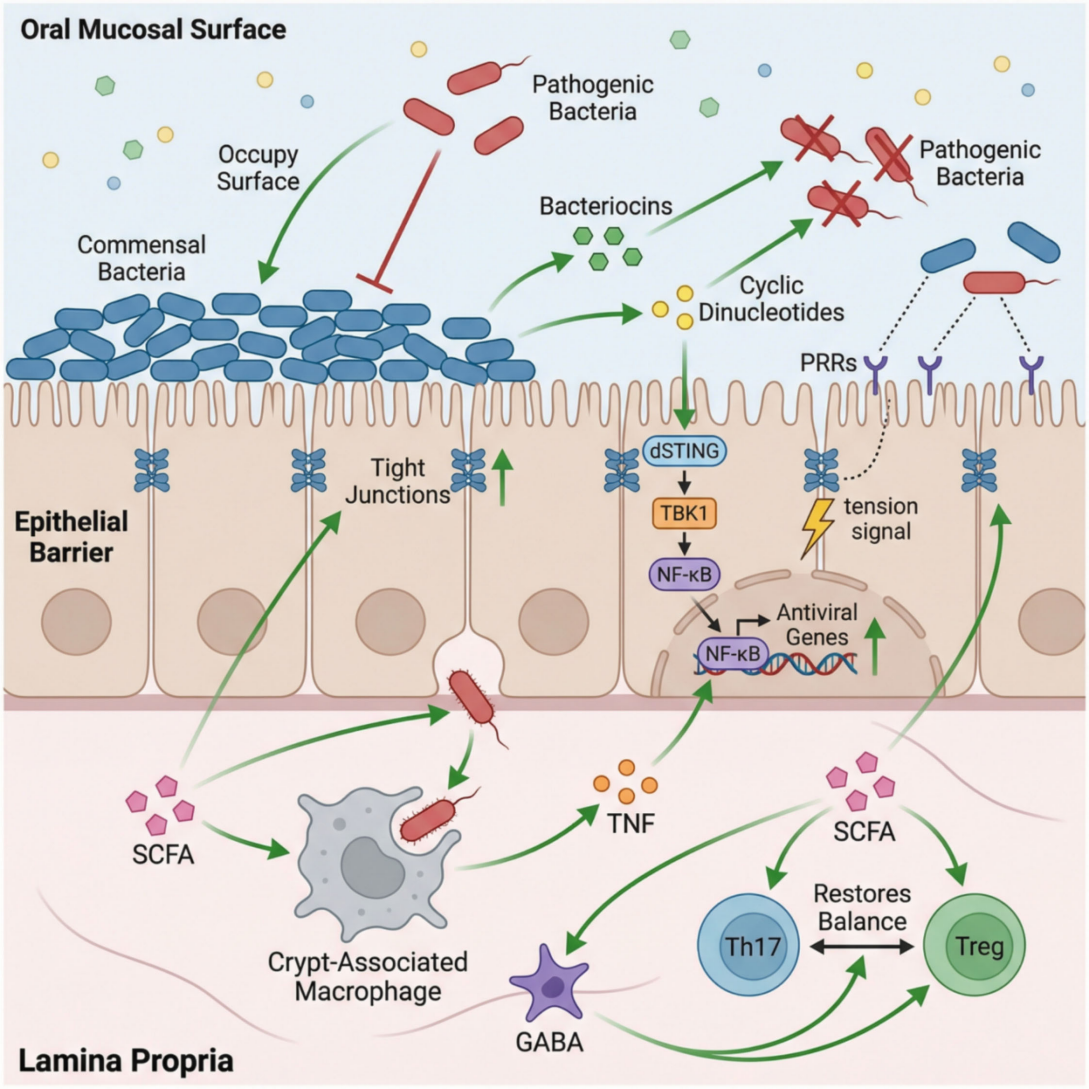
Commensal bacteria-mediated regulation of the oral mucosal epithelial barrier and the network for maintaining local immune homeostasis.

Commensal bacteria colonizing the oral cavity form the first line of defense against invading pathogenic bacteria. Under normal physiological conditions, commensal bacteria can block the adhesion of pathogens by occupying mucosal surface niches, and limit their growth and proliferation by competing for nutrients such as carbon and nitrogen (33). Furthermore, many oral commensals produce bactericidal substances such as bacteriocins that directly kill or inhibit latent pathogenic bacteria in the surrounding microenvironment (34). Rather than functioning merely as passive occupants, resident oral communities continuously shape epithelial antimicrobial tone and ecological resistance through locally delivered microbial ligands and metabolites, thereby constraining pathogen overgrowth while preserving tissue compatibility (34–36). This biological barrier established by the resident microbiota plays a vital role in maintaining the stability of the oral microenvironment and preventing infection (**Figure 2**).

The interactions between commensal bacteria and host epithelial and immune cells are crucial for maintaining the epithelial barrier function and educating the innate immune system. Oral epithelial cells are continuously exposed to a large number of microbe-associated molecular patterns (MAMPs), which are detected by the PRRs on their surface (35). This constant, low-level stimulation – termed “tonic signaling” – is indispensable for the homeostatic function of epithelial cells. The presence of tonic signaling enables long-term maintenance of host-microbiota interactions in healthy oral tissue models, preserving host barrier integrity and the crosstalk between healthy microbial populations (36). This interaction helps “train” the local immune system to maintain a state of moderate alertness, allowing it to respond to real threats while avoiding excessive inflammatory reactions. For instance, intercrypt macrophages in the lamina propria act as sentinel cells: upon sensing Gram-negative bacteria that have crossed the epithelium, they release TNF, which modulates the NF-κB defense response of adjacent epithelial cells, thereby eliciting an effective and controlled defense (37). This surveillance mechanism modulated by commensal bacteria is critical for maintaining the integrity of the mucosal immune barrier in the gingiva.

The metabolites of the oral microbiota also mediate host-microbiota interactions and indirectly consolidate barrier function. For instance, short-chain fatty acids (SCFAs) not only serve as an energy source for epithelial cells but also directly enhance epithelial barrier integrity by upregulating the expression of tight junction proteins (38). SCFA-associated signaling has been implicated in the maintenance of epithelial homeostasis and restoration of the Th17/Treg balance during oral bone tissue repair, suggesting that microbial metabolites actively shape barrier-repair competence (38). Furthermore, SCFAs modulate myeloid and lymphoid responses through G protein-coupled receptor signaling, although much of the mechanistic insights have been derived from non-oral mucosal systems and require direct validation in oral tissues (39). Other microbial metabolites such as γ-aminobutyric acid (GABA) also participate in the regulation of local immunity and homeostasis (40). These microbial metabolites form an intricate chemical signaling network that indirectly and effectively strengthens the oral mucosal barrier through multiple pathways, including modulation of epithelial metabolism, enhancement of intercellular connections, and shaping of the immune landscape.

## 3 Core of barrier function remodeling: the epithelial-immune-microbial interaction network

### 3.1 Epithelial cells as immune sentinels and coordinators

**Figure 3:**
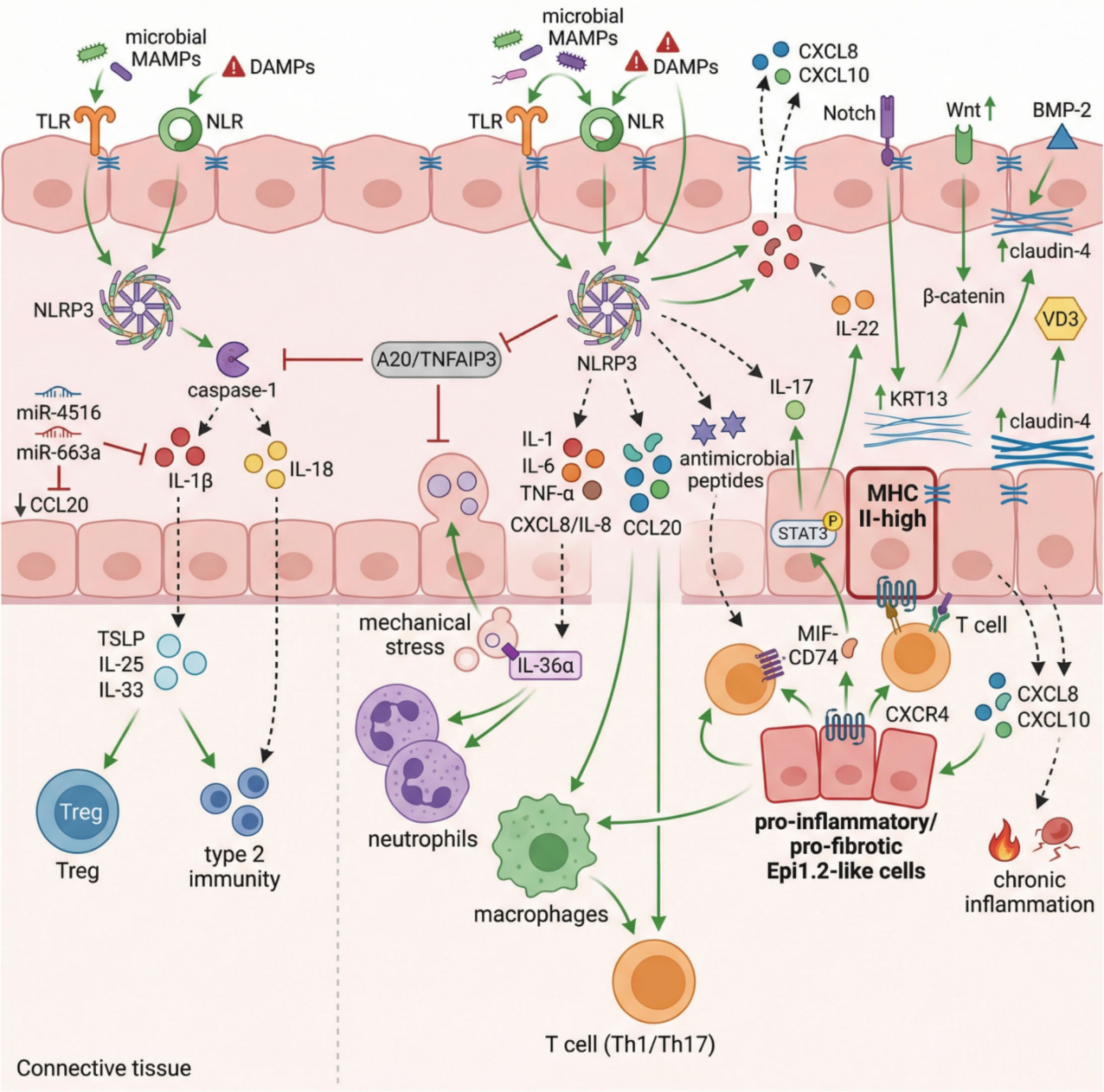
NLRP3 inflammasome-mediated imbalance of oral mucosal epithelial immune responses and the mechanism of chronic inflammation.

Oral epithelial cells are not only part of a physical barrier, but also function as active immune sentinels and coordinators. They express multiple PRRs, including members of the Toll-like receptor (TLR) and NOD-like receptor (NLR) families, to sense and respond to MAMPs and damage-associated molecular patterns (DAMPs) (41) (**Figure 3**). The NLRP3 inflammasome, an intracellular receptor expressed in oral mucosal epithelial cells, is activated upon sensing pathogenic or danger signals, and promotes activation of caspase-1, leading to the production and release of potent proinflammatory cytokines such as IL-1β and IL-18 (42). This sensing process forms the basis of initiating local immune responses. Activated epithelial cells secrete a range of cytokines (IL-1, IL-6, TNF-α) and chemokines (CXCL8, CCL20), as well as AMPs, to recruit and shape the responses of immune cells (41). For example, gingival epithelial cells infected with *Porphyromonas gingivalis* induce secretion of IL-6 and IL-8 (CXCL8), a response that is tightly controlled by regulators such as A20 (TNFAIP3) (43). Additionally, epithelial cells secrete IL-36α-containing extracellular vesicles in response to mechanical trauma and other stress to enhance immune signal transduction (44), thus forming the body’s first active barrier against invading pathogenic bacteria.

The activation of NLRP3 inflammasome in oral epithelial cells is a coordinated two-step process. A priming signal is first initiated when microbial ligands or endogenous DAMPs engage PRRs, thereby activating NF-κB and inducing the transcriptional upregulation of NLRP3, pro-IL-1β, and pro-IL-18. A second activation signal is then provided by epithelial stress responses such as ROS accumulation, potassium efflux, mitochondrial dysfunction, lysosomal destabilization, ATP-related danger signaling, or mechanical injury, which promote inflammasome assembly, caspase-1 activation, and the maturation of IL-1β and IL-18 (42, 44). In the short term, this inflammatory axis strengthens antimicrobial surveillance and leukocyte recruitment; however, excessive or unresolved inflammation amplifies epithelial injury, destabilizes junctional complexes, increases paracellular permeability, and shifts the local response to self-sustaining barrier damage (41, 42).

Oral epithelial cells exhibit highly plastic immunoregulatory functions that drive opposing immune responses. Under homeostatic conditions, epithelial cells release “alarmines” such as thymic stromal lymphopoietin (TSLP), IL-25 and IL-33, which tend to drive type 2 immune responses and Treg cell differentiation, thereby facilitating the induction of immune tolerance to food antigens and commensal microorganisms (45). This tolerance is critical for preventing excessive inflammation in response to a large number of harmless antigens. However, upon pathogenic infection, the epithelial cells drive Th1- and Th17-dominant immune responses to effectively eliminate the pathogens (45). For example, epithelial cells recognize *Candida albicans* and initiate a cytokine cascade during oral candidal infection. IL-22, a cytokine secreted by epithelial cells, stimulates the growth of oral basal epithelial cells and maintains barrier integrity by activating STAT3, while allowing the overlying spinous layer epithelial cells to respond to IL-17 and exert antibacterial activity (46). This spatially distinct yet cooperative interaction between IL-22 and IL-17 exemplifies mobilization of adaptive immunity by the epithelial cells to clear specific pathogens; furthermore, epithelial cells can inhibit production of the chemokine CCL20 by inducing specific miRNAs (miR-4516 or miR-663a) (47), thereby balancing inflammatory and antibacterial responses to different oral bacteria (*Streptococcus gordonii*) and preventing excessive immune reactions.

These molecular circuits can also be mapped onto defined oral disease phenotypes, which strengthens their translational relevance. In recurrent aphthous stomatitis, epithelial danger sensing appears to be skewed toward an ulcer-promoting state, as recurrent lesions have been linked to altered TLR responsiveness and NLRP3-associated genetic susceptibility. This supports the concept that exaggerated PRR-inflammasome signaling may convert otherwise self-limited epithelial injury into recurrent ulcerative inflammation (48, 49). In oral lichen planus, epithelial alarmins are more directly connected to chronic interface mucositis: TSLP/TSLPR signaling promotes dendritic cell (DC)-T-cell crosstalk, IL-25 is associated with erosive disease severity and persistent type 2 amplification, and IL-33 expression in lesional tissues is consistent with ongoing epithelial stress and immune activation (50–52). In radiation- or chemotherapy-induced mucositis, therapy-related DAMP release and microbial perturbation engage PRR/NF-κB/IL-1β cascades, while mitochondrial stress can couple to NLRP3 activation, thereby intensifying epithelial apoptosis, ulceration, pain, and delayed restitution (53, 54). Moreover, in pre-neoplastic inflammatory contexts such as oral lichen planus with dysplastic tendency and oral leukoplakia undergoing malignant transition, persistent epithelial alarm signaling and cytokine-rich myeloid remodeling may help establish an inflammatory field that favors immune dysregulation, stromal support, and stepwise progression toward OSCC (11, 55).

Multiple signaling pathways regulate the proliferation and differentiation of epithelial cells, as well as the expression of barrier-related genes, which shape their functional state and the interaction with the immune system. Evolutionarily conserved signaling pathways such as Notch and Wnt/β-catenin play a central role in determining epithelial cell fate and maintaining homeostasis (56). For example, keratin 13 (KRT13) is a key factor for maintaining oral epithelial integrity; mice lacking KRT13 exhibit a leukoplakia-like neoplastic phenotype and dysregulation of genes associated with epithelial differentiation, immunity and stress-activated kinase signal transduction, which impairs cell growth and differentiation (56). Other studies have suggested that bone morphogenetic protein 2 (BMP-2) may also be involved in the regulation of oral mucosal keratinization by directly promoting epithelial cell differentiation and proliferation (57). The barrier function of epithelial cells is closely linked to intercellular junctions, particularly the tight junctions (TJs) formed by the claudin protein family. Vitamin D3 (VD3) can increase the level of claudin-4 in gingival epithelial cells and enhance transepithelial electrical resistance (TEER), resulting in stronger physical barrier (30). Upon barrier disruption, epithelial cells actively recruit immune cells by secreting cytokines and chemokines (CXCL8, CXCL10) (58), a phenomenon commonly observed in chronic inflammatory diseases. In addition, single-cell transcriptomic studies have identified a subset of epithelial cells with high expression of major histocompatibility complex class II (MHC II) molecules, along with a unique population of proinflammatory and profibrotic epithelial cells (Epi1.2 in oral submucous fibrosis). These cell subsets drive local immunopathological processes through receptor-ligand interactions (MIF-CD74, CXCR4 signaling) with immune cells such as T cells (10). Thus, epithelial cells integrate signals from the microenvironment and regulate their own functional state, acting as key regulators of the recruitment and activation of underlying immune cells.

NF-κB and STAT3 are the two major hubs that translate microbial and immune cues into epithelial programs of defense or repair. NF-κB is activated downstream of TLR/NLR signaling and inflammatory mediators such as TNF-α and IL-1β, and induces chemokines, AMPs, and inflammasome-related transcripts that mobilize innate immunity. By contrast, epithelial STAT3 is mainly activated by IL-22 and IL-6 family cytokines, and promotes the survival, proliferation, and metabolic adaptation of epithelial cells, which aid in the restitution of damaged mucosal surface (46). The biological outcome depends less on the activation of either pathway alone than on their timing, magnitude, and balance: restrained NF-κB activity coupled with appropriately timed STAT3 signaling can support controlled antimicrobial defense followed by re-epithelialization, whereas sustained NF-κB activation along with aberrant STAT3-driven survival signaling may perpetuate chronic inflammatory loops, alter differentiation programs, and favor fibro-inflammatory remodeling (30, 46, 58). In parallel, cytokine networks centered around IL-17, IL-22, TSLP, IL-25, and IL-33 further tune this balance by coupling epithelial sensing to neutrophil recruitment, AMP production, immune tolerance, or type 2 polarization according to local cues (47).

### 3.2 Immediate response of innate immune cells in barrier remodeling

**Figure 4:**
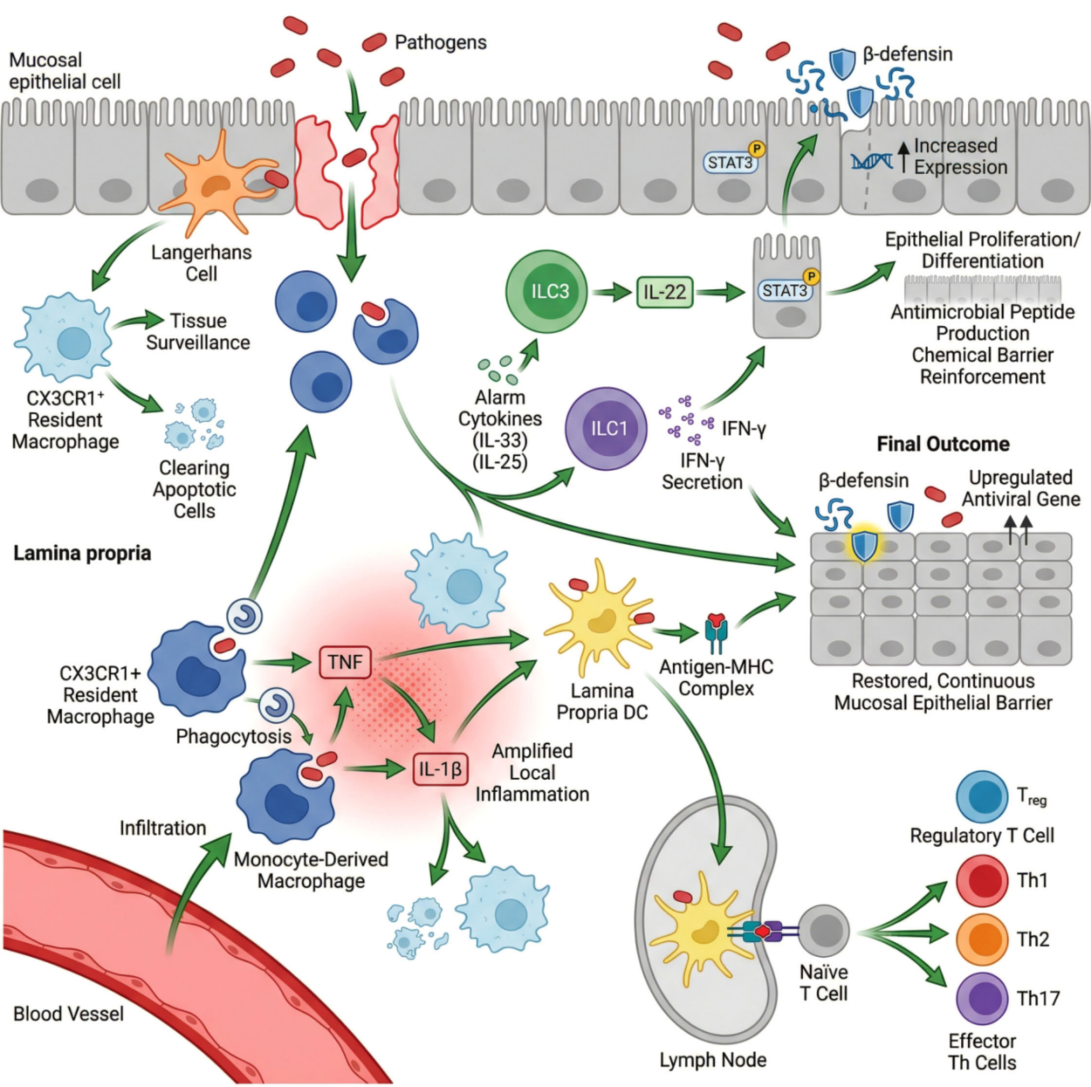
Mechanisms of local inflammatory responses mediated by oral mucosal immune cells and the regulation of epithelial barrier repair upon pathogenic invasion.

Innate immune cells form the first line of defense for mucosal barrier function. Following pathogenic microbial invasion or tissue damage, they mediate the early phases of barrier remodeling triggered through phagocytosis, antigen presentation and the release of various cytokines (**Figure 4**). The macrophages in the mucosal lamina propria are highly heterogenous, and their functions are associated with their origin. Resident CX3CR1+ macrophages perform constant tissue surveillance, clear apoptotic cells and maintain tissue homeostasis (3). However, in the presence of inflammation, monocyte-derived macrophages extensively infiltrate the infected or damaged site, where they clear pathogens and cellular debris through phagocytosis, and secrete large amounts of TNF and IL-1β to amplify local inflammatory responses for subsequent tissue repair (59). This transition from homeostatic surveillance to inflammatory response is a key feature of macrophages in mediating early barrier remodeling.

DCs act as a bridge between innate and adaptive immune responses and determine the direction of immune reactions (tolerance or immunity) (6). There are two main subsets of DCs in the oral mucosa: Langerhans cells in the epithelium and DCs in the lamina propria, both of which efficiently capture antigens from oral microorganisms or damaged tissues. Antigens captured by DCs are processed into peptide fragments, which are presented to naive T cells after the maturation of DCs and their migration to draining lymph nodes (60). DCs in homeostatic or tolerogenic milieus induce the differentiation of naïve T cells to regulatory T cells (Tregs) cells, which maintains immune tolerance to commensal bacteria and even harmless antigens. In contrast, upon pathogenic invasion, DCs tend to drive the generation of effector T cell responses (Th1, Th2 or Th17) to eliminate the infection. Therefore, the function of DCs dictates the direction of immune microenvironment remodeling after acute barrier disruption.

Innate lymphoid cells (ILCs) are a subset of innate immune cells lacking specific antigen receptors. They rapidly respond to alarmins (IL-33, IL-25) in the tissue microenvironment and secrete effector cytokines, and therefore play a central role in barrier defense and repair (61). Group 3 ILCs (ILC3s) secrete IL-22, which repairs epithelial barrier damage caused by infection or injury by stimulating the proliferation and differentiation of epithelial cells via STAT3 activation (62). It also induces the synthesis of β-defensins by epithelial cells to enhance the mucosal chemical barrier and limit further pathogenic colonization and invasion. On the other hand, ILC1s maintain the “anti-viral alert state” of the oral mucosa by constitutively producing low levels of interferon-γ (IFN-γ), which upregulates multiple anti-viral genes in epithelial cells for potential viral invasion (63). Thus, ILCs are the core population linking innate immune signals to epithelial barrier repair functions. Under physiological or adaptive remodeling, these circuits are typically transient, proportionate, and self-limited; under pathological remodeling however, they may stabilize into a chronic inflammatory niche that couples epithelial stress, immune reprogramming, and microbial dysbiosis, thereby driving maladaptive barrier deterioration rather than effective restoration.

These signaling pathways are distributed across, and reinforced by, the broader epithelial-immune-microbial network rather than being confined to epithelial cells alone. Macrophage-derived TNF and IL-1β can further enhance epithelial NF-κB activity and inflammasome priming, while DCs translate epithelial-derived alarm signals into T cell polarization programs, and ILC3-derived IL-22 activates epithelial STAT3 to restore barrier gene expression and regenerative capacity (62). At the ecological level, microbiota-derived metabolites such as SCFAs help support junctional integrity and immune restraint, whereas dysbiosis shifts signaling toward persistent IL-1β, TNF-α and IL-6 production, and a Th17-skewed inflammatory microenvironment. Therefore, signaling events during oral mucosal barrier remodeling should be viewed as feed-forward and feedback circuits linking epithelial sensing, innate immune amplification, adaptive immune programming, and microbial ecological change, rather than as isolated molecular events.

### 3.3 Barrier dysfunction and tumor microenvironment remodeling in oral squamous cell carcinoma

Persistent impairment of the oral mucosal barrier creates a permissive niche for the initiation and progression of oral squamous cell carcinoma (OSCC). Recurrent epithelial injury, unresolved inflammation, and defective junctional integrity facilitate sustained exposure of basal and suprabasal epithelial compartments to microbial products, reactive oxygen species (ROS), proteases, and pro-inflammatory cytokines. Under these conditions, the NF-kB/STAT3 pathway, along with IL-1β-, IL-6-, TNF-α-, and CXCL8-mediated circuits, promotes epithelial survival, proliferation, partial epithelial-mesenchymal transition (EMT), angiogenesis, and matrix remodeling, thereby linking chronic mucosal inflammation to malignant transformation and invasive behavior (7). Recent clinicopathological evidence suggests that stromal activation markers and extracellular matrix (ECM) remodeling factors in oral cancer are associated with reduced E-cadherin levels, increased vimentin/podoplanin expression, higher neutrophil accumulation, and diminished intratumoral CD8+ T cell infiltration, which are consistent with a barrier-disrupted and immune-reprogrammed invasive niche (64).

From the perspective of barrier remodeling, OSCC should therefore be viewed not only as an epithelial malignancy but also as the consequence of reciprocal epithelial-stromal-immune reorganization. Cancer-associated fibroblasts (CAFs), myeloid cells, and dysregulated epithelial cells jointly remodel the local barrier by depositing and reorganizing the ECM, increasing vascular permeability, amplifying chemokine gradients, and establishing immunosuppressive feedback loops, and these processes can be exacerbated by tumor-derived mediators. For example, OSCC-derived GDF15 promotes the transformation of fibroblasts and bone marrow-derived mesenchymal stromal cells into CAFs, whereas CAF-derived GDF15 enhances oxidative stress, chronic inflammatory signaling, and neutrophil infiltration in head and neck squamous cell carcinoma (HNSCC) through PI3K/AKT/STAT3-related programs (65, 66). These observations suggest that barrier dysfunction, stromal activation, and innate immune skewing are mechanistically intertwined during oral tumor progression.

Emerging evidence also indicates that microbial dysbiosis is an active driver of tumor microenvironment (TME) remodeling rather than a passive bystander. Periodontal pathogens and intratumoral bacteria can promote oncogenic signaling, weaken epithelial barrier-related programs, and reshape antitumor immunity. *Fusobacterium nucleatum* is known to induce DNA damage and enhance proliferation of oral cancer cells through the Ku70/p53 pathway (67), whereas *P. gingivalis* promotes the proliferation of OSCC cells through the miR-21/PDCD4/AP-1 axis (8). Furthermore, *Parvimonas micra* is enriched in OSCC tissues and metastatic lesions, and drives metastasis by engaging the TmpC-CKAP4 axis, thereby activating HIF-1α-dependent glycolysis, autophagy, and CAF/TAM-associated remodeling programs (9). Likewise, a 2025 OSCC cohort study demonstrated that high-risk intratumoral microbial signatures were associated with EMT, depletion of CD8+ T cells, infiltration of M0 macrophages, upregulation of TGF-β1/CD276, and T cell exclusion, all of which support a microbiota-immune axis in immune evasion (11). Notably, preclinical evidence also suggests that therapeutic modulation of the oral microbiome can reshape the OSCC immune milieu and enhance response to PD-1 blockade (12). Together, these findings support a model in which chronic inflammation, microbial dysbiosis, and immune dysregulation cooperate to convert barrier remodeling from a reparative program into a tumor-promoting process.

## 4 Fine regulation and memory of barrier function by adaptive immunity

### 4.1 Mucosa-associated lymphoid tissue and local antibody responses

**Figure 5:**
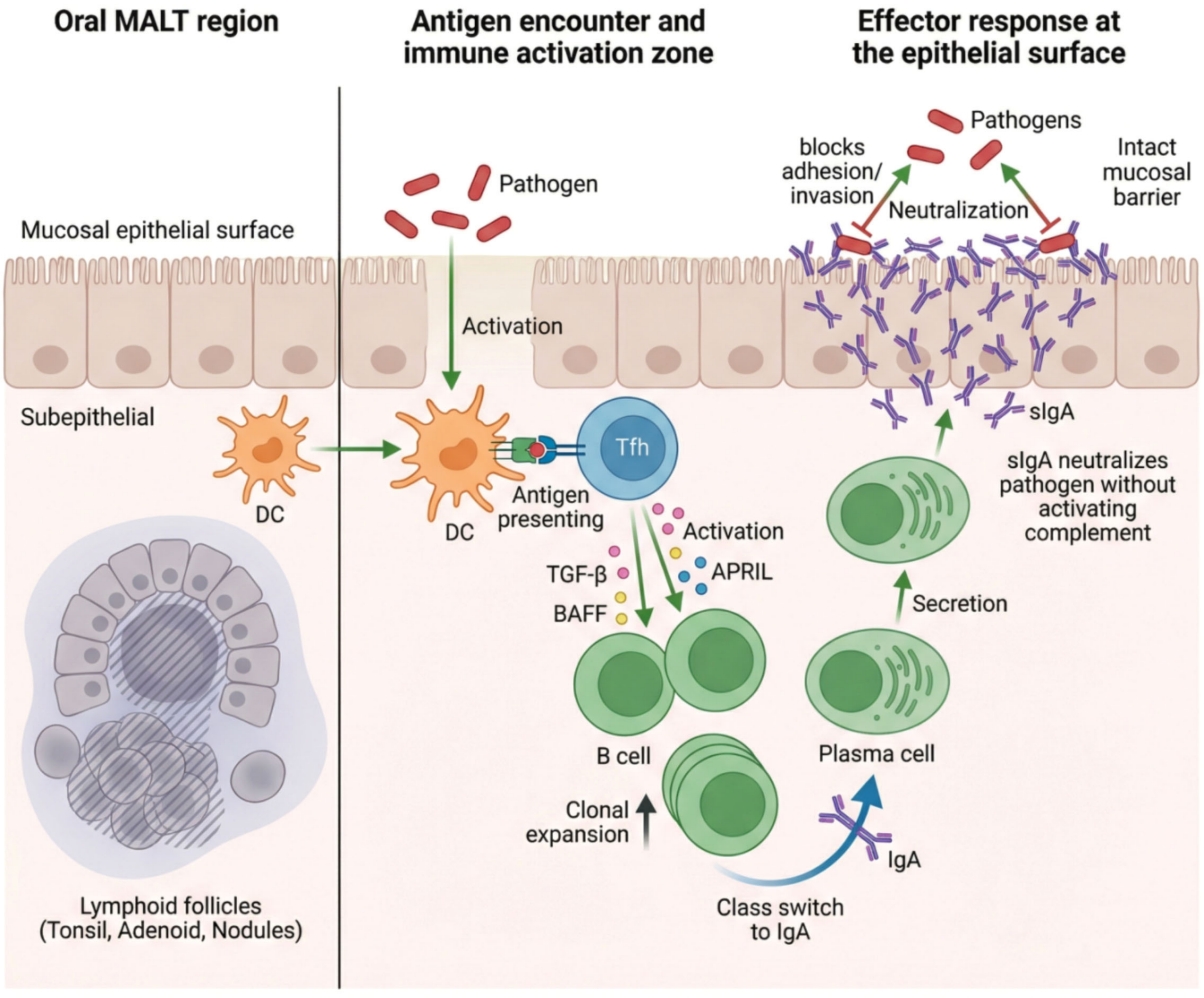
Antigen presentation and IgA class switching in the oral MALT region: the sIgA-driven immune defense mechanism of the oral mucosal barrier.

Mucosa-associated lymphoid tissue (MALT) is the primary site for generating specific immune responses at mucosal sites. Oral MALT includes tonsils, adenoids and scattered lymphoid nodules throughout the oral cavity. These tissues are the sites for the aggregation, activation and proliferation of B and T lymphocytes following pathogenic invasion through the oral route. For example, in fish infected with parasites, local aggregation and proliferation of B and T cells can be observed in MALTs such as the gills and head kidney (68). These dynamic changes in local lymphocytes drive the production of highly efficient and specific mucosal antibodies, ensuring that immune responses can be rapidly initiated at the site of invasion without complete reliance on the systemic immune system (**Figure 5**).

Secretory IgA (sIgA) is the major antibody isotype present on the oral mucosal surface, and is essential for maintaining mucosal barrier homeostasis and defensive function. One of the key mechanisms of sIgA is “immune exclusion”: it neutralizes pathogenic antigens, such as viruses, bacteria and their toxins, and prevents their adhesion to epithelial cells, thus clearing the pathogens before they invade the underlying tissues. Unlike IgG and other immunoglobulins, the activation of sIgA does not typically trigger significant inflammation or complement cascade reactions, thus avoiding self-damage caused by excessive inflammatory responses. In the fish mucosal immune system, this function is mediated by mucosa-specific immunoglobulins such as IgT, which is elevated in mucosal secretions after infection (68). This underscores the fact that the local production of specific antibodies by the mucosa is crucial for forming an effective first line of defense. The development of vaccines against mucosal pathogens is precisely aimed at inducing such local antibody responses. For example, oral vaccines encapsulating antigens in hydrogel microspheres can effectively deliver antigens to intestinal MALT and significantly increase the levels of specific antibodies such as IgM in mucosal tissues to enhance protection (69).

Apart from serving as a classical mediator of immune exclusion, the local B-cell/plasma-cell axis also stabilizes the oral mucosal barrier by shaping microbial ecology in a selective manner. The sIgA produced by plasma cells in the salivary glands and mucosal lamina propria can agglutinate microorganisms, mask microbial adhesins, neutralize enzymes and toxins, and reduce direct epithelial contact without necessarily eliminating commensal colonizers. This function is particularly important in the oral cavity, where tooth-associated biofilms, continuous salivary flow, and repeated exposure to dietary and environmental antigens require immune containment rather than indiscriminate microbial clearance. In this context, antibody coating acts as a non-inflammatory filtering system that contributes to oral microbial homeostasis and reinforces tolerance to commensal microorganisms, while preserving the capacity to restrain ecological expansion of pathobionts (70, 71).

Evidence further indicates that impairment of salivary sIgA-mediated immunity may increase periodontal susceptibility and weaken antifungal defense. In IgA-deficient mice, defective production of salivary sIgA is associated with oral dysbiosis and enhanced alveolar bone loss, supporting a causal link between local antibody insufficiency, microbial imbalance and periodontal tissue destruction (72). Conversely, experimental periodontitis induces expansion of IgA-producing B cells in salivary glands following oral dysbiosis, suggesting that the B-cell/plasma-cell axis functions as an adaptive module that attempts to re-establish ecological control under inflammatory stress (73). Mucosal IgA also limits the overgrowth of commensal *C. albicans* at sites of colonization, and helps prevent the transition from controlled carriage to dysbiosis-associated mucosal disease (74). Taken together, these findings indicate that local antibody-mediated immunity is not merely an accessory effector arm, but a central regulator of barrier stability that integrates immune tolerance, ecological containment and pathogen-specific protection in the oral mucosa (73, 75).

The secretion of sIgA is a precise and ordered process involving the coordinated action of DCs, T cells and B cells, as well as specific cytokines in the local microenvironment. First, DCs beneath the mucosal epithelium capture and process antigens, then present them to T follicular helper (Tfh) cells. The activated Tfh cells provide costimulatory signals to B cells and secrete cytokines to support their clonal expansion, class switching and affinity maturation in the lymphoid follicles of MALT. Transforming growth factor-β (TGF-β) produced by the epithelial and stromal cells is the major cytokine that promotes antibody class switching to IgA. In addition, members of the TNF superfamily, B cell-activating factor (BAFF) and a proliferation-inducing ligand (APRIL), also contribute to the survival, proliferation and differentiation of B cells into plasma cells at mucosal sites. Studies have shown that the local proliferation of B cells at mucosal sites is critical for the rapid generation of antibodies. For example, immune stimulation in carp induces massive expansion of B cells coexpressing immunoglobulin and proliferating cell nuclear antigen (PCNA) beneath the gill mucosal epithelium (76), suggesting that B cells can recognize corresponding antigens and proliferate locally at this site to rapidly increase the number of antibody-secreting cells. This mode of antibody production, mediated by local cellular cooperation and cytokine secretion, ensures the specificity and effectiveness of the sIgA response for maintaining the oral mucosal defense function.

### 4.2 Barrier patrolling function of tissue-resident memory T cells

Tissue-resident memory T (TRM) cells are an important component of immune memory; they strategically reside in all barrier tissues including the oral mucosa to provide the first line of defense against infection and cancer (77). After antigen encounter, CD8+ and CD4+ TRM cells persist long-term in the oral epithelium and lamina propria without recirculating in the lymphatic system, thus forming a stable local immune sentinel force (78). After re-encountering the same pathogen, these cells mount an extremely rapid response and quickly release effector cytokines (IFN-γ and IL-17) to initiate innate and adaptive immune responses, and strengthen local barrier defense (78). This rapid response capability allows them to directly attack pathogens without the need to recruit circulating memory T cells (79). Studies in non-human primate models have demonstrated that systemic vaccination can induce TRM cells in barrier tissues including the mucosa; upon antigen stimulation, TRM cells activate local stromal and parenchymal cells, as well as innate and adaptive immune cells, thereby amplifying alarm signals and initiating multiple types of host defense mechanisms (80). Thus, TRM cells are key effector cells for effective and rapid immune surveillance and response in the oral mucosa.

**Figure 6:**
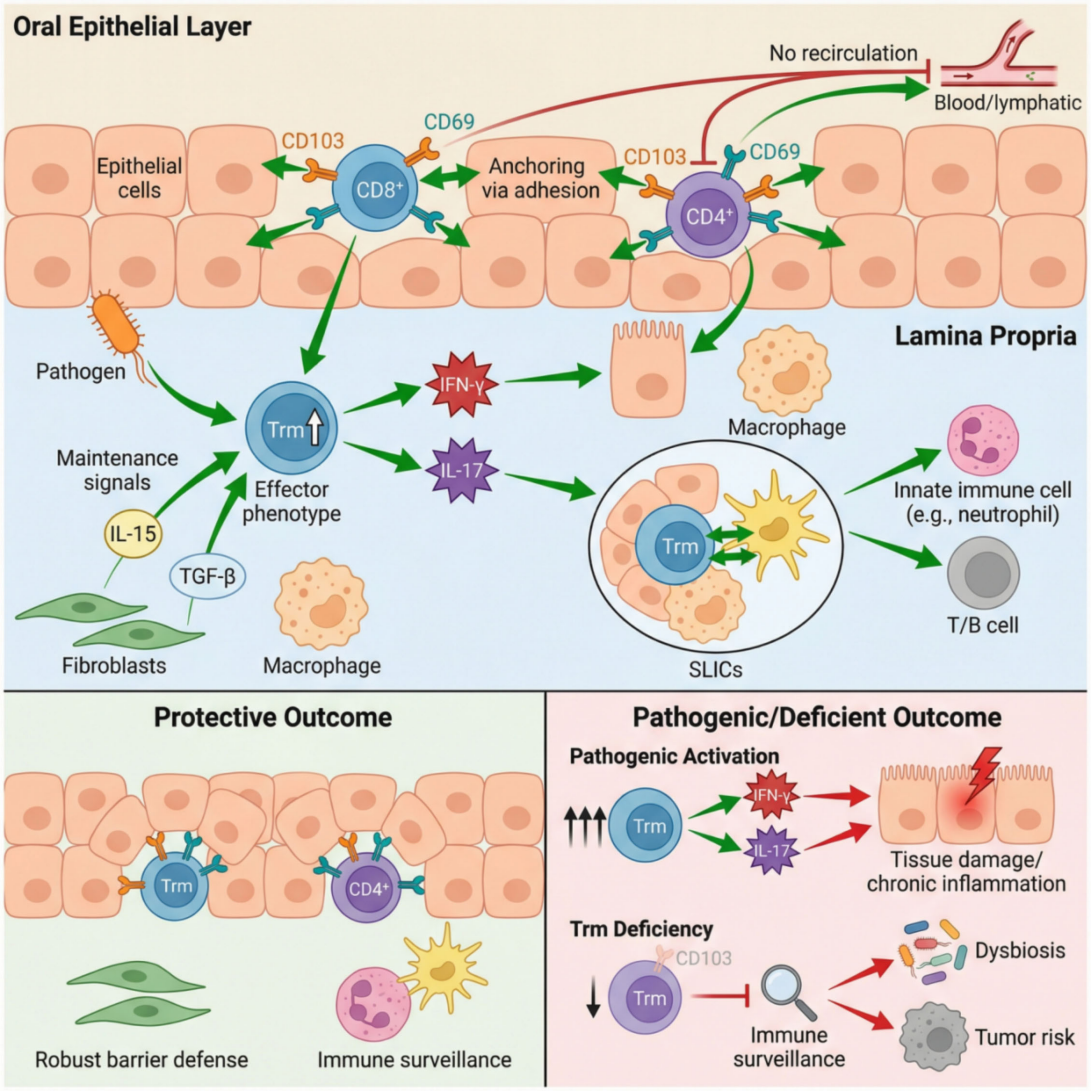
Anchoring and bidirectional immunoregulation of TRM cells in the oral epithelium: the association between the defensive function of the oral mucosal barrier and pathological risks.

The long-term survival and functional maintenance of TRM cells depend on close interactions with the surrounding tissue microenvironment, particularly with stromal cells such as epithelial cells and fibroblasts (81), which together form a long-term effective local immune surveillance system (**Figure 6**). The survival and retention of TRM cells are precisely regulated by the local cytokine network, with IL-15 and TGF-β playing key roles (82). For example, macrophage-derived IL-15 in the ascites of patients with liver cirrhosis can imprint and maintain a TRM-like phenotype in CD8+ T cells via bystander activation, preserving their potent cytotoxicity and proinflammatory response capacity even after the resolution of inflammation (82). In addition, TRM cells express specific adhesion molecules (CD103 and CD69) that anchor them to epithelial cells, ensuring their tissue-resident properties (81). Studies have identified unique sublingual immune clusters (SLICs) in the oral mucosa, where DCs and T cells (including TRM cells) are in close contact, and form a microenvironment that promotes intercellular interactions. This structure expands under inflammatory conditions and strengthens the local immune surveillance network (83). Thus, TRM cells do not exist in isolation but are part of a dynamic network composed of cytokines, intercellular contacts, and specific tissue structures.

More direct evidence in recent years has further elucidated the relevance of TRM cells within the oral mucosa. Single-cell and immune-repertoire studies of oral lichen planus lesions have identified activated CD8+ TRM populations and linked them to epithelial damage through IFN-γ-, TNF-α-, and IL-17-associated cytokine networks. This supports a functional as opposed to a mere bystander role for TRM cells in chronic interface inflammation (12, 13, 84). These findings suggest that persistent local antigenic stimulation may “lock” tissue-retained cytotoxic T cells into a self-sustaining inflammatory circuit that contributes to chronic oral mucosal injury. At the same time, oral TRM cells are not uniformly pathogenic. Experimental studies have shown that bona fide oral CD103+ TRM cells are distributed across the tongue, gingiva, palate, and buccal mucosa, and can rapidly trigger local innate-alert and protective recall programs after antigen re-encounter, consistent with a specialized role in oral mucosal immune surveillance (85). However, the same sentinel positioning may become deleterious in chronic inflammatory oral lesions: reactivation of mouth-resident antiviral CD8+ TRM cells aggravates experimental periodontitis and amplifies gingival inflammation and alveolar bone loss, indicating that TRM cells can shift from protective effectors to drivers of persistent local tissue injury under dysbiotic or inflammatory conditions (86). In oral tumors such as HNSCC, higher TRM signatures and CD103+ T cell infiltration correlate with an inflamed TME and more favorable prognosis, which is consistent with an anti-tumor surveillance function; however, prolonged antigen exposure may also promote dysfunctional or exhausted states that limit durable protection (87).

Despite their protective effects, TRM cells can contribute to chronic inflammation when dysregulated or abnormally activated (77). For example, TRM cells in the oral cavity may be one of the pathogenic factors in certain diseases such as oral lichen planus (88). The long-term presence of TRM cells in the body is a two-edged sword: these cells perform patrolling and surveillance under normal conditions, but can exert either protective or antagonistic effects in a pathological state. For instance, the number of CD103+ CD8+ TRM cells and γδ T cells in the is significantly reduced in the colonic mucosa of patients with familial adenomatous polyposis and colorectal cancer (89). The impaired immune surveillance function is associated with microbial dysbiosis and an increased risk of tumorigenesis (90), suggesting that defective TRM cell function may lead to barrier dysfunction and tumor immune escape. Conversely, under certain chronic inflammatory conditions, TRM cells may be persistently activated and secrete excessive proinflammatory cytokines (IFN-γ, IL-17), thereby driving tissue damage and disease chronicity (91). TRM cells are also recognized as key participants in regulating adaptive immune responses in allergic and autoimmune diseases (92). Thus, an in-depth understanding of the precise regulatory mechanisms of TRM cells in oral health and disease is crucial for the development of targeted intervention strategies that enhance their protective functions while inhibiting their pathogenic potential (81).

## 5 Interactions between systemic diseases and the oral mucosal barrier

### 5.1 Negative impact of metabolic diseases on barrier function

**Figure 7:**
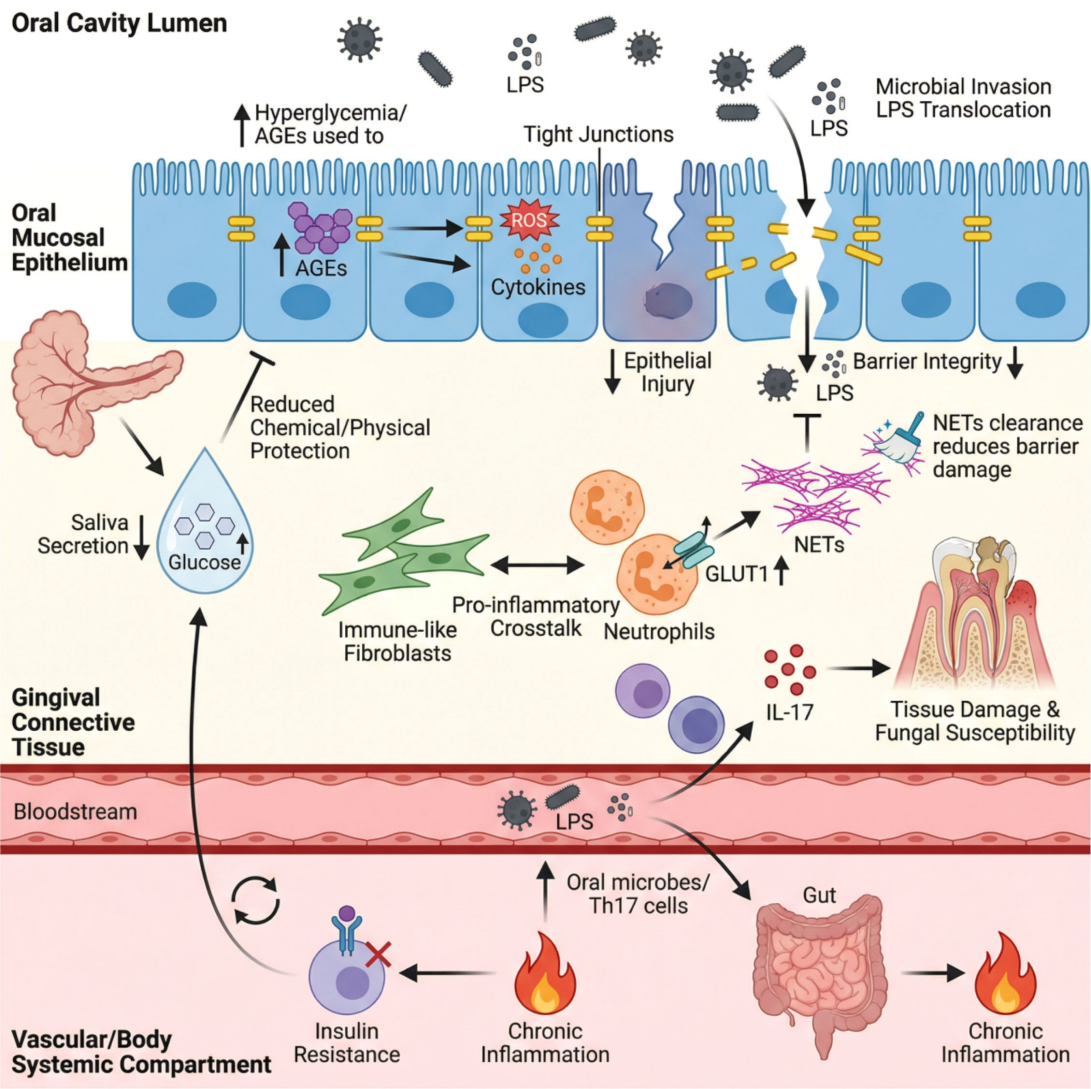
Mechanisms of oral mucosal barrier damage mediated by hyperglycemia/AGEs.

Hyperglycemia is one of the key pathological processes that impairs oral mucosal barrier function in diabetes (93) (**Figure 7**). Long-term exposure to high glucose levels induces the accumulation of advanced glycation end products (AGEs) in epithelial cells, and the overproduction of ROS and proinflammatory cytokines, which cause epithelial damage. Epithelial cell injury further impairs the integrity of tight junctions, which are essential for maintaining mucosal barrier function. Single-cell transcriptomic sequencing studies have provided direct evidence for this hypothesis: the gingival tissues of mice with type 2 diabetes (T2D) show reduced epithelial/stromal cell ratio and impaired barrier function (94). Epithelial injury progresses from structural barrier disruption to broader barrier dysfunction, rendering the oral mucosa more susceptible to microbial attack, leading to persistent local inflammation. Furthermore, inflammatory signaling pathways are activated in diabetic gingivitis, and the fibroblast-neutrophil crosstalk is the driving force for the accompanying periodontal tissue destruction (94). “Immune-like” stromal cells, mainly fibroblasts, exhibit immune hypersensitivity and are associated with the recruitment of bone marrow-derived cells, which also contribute the dysregulated innate immune homeostasis in diabetic gingiva (94).

Salivary gland dysfunction in diabetic patients reduces saliva secretion and increases glucose levels in the saliva (95), which significantly impairs its chemical and physical barrier functions, and increases the risk of mechanical trauma and microbial colonization. On the other hand, neutrophils infiltrating the gingival mucosa under hyperglycemic conditions undergo metabolic reprogramming characterized by the upregulation of glucose transporter 1 (GLUT1), and increased glucose uptake and glycolysis rates (93). This abnormal metabolism leads to the excessive release of neutrophil extracellular traps (NETs), and the clearance of these NETs can alleviate mucosal barrier damage (93). In fact, NET levels are significantly elevated in local gingival lesions of patients and animal models with T2D, and correlate with the clinical severity of the disease (64). This functional alteration of neutrophils, combined with impaired salivary barrier function, makes the oral mucosa prone to infection and delays wound healing. Clinical observations have also shown that excessive IL-17 activity in diabetic patients increases the risk of periodontal tissue destruction and fungal infection (95).

Oral mucosal barrier injury may also induce or exacerbate systemic metabolic abnormalities. Oral microorganisms and their metabolites, such as lipopolysaccharides, can enter systemic circulation through the damaged mucosa, causing chronic, low-grade systemic inflammation (96), which is a major driver of insulin resistance. Studies have shown an inextricable interaction between intestinal microbial dysbiosis, intestinal barrier injury, chronic systemic inflammation and abnormal energy metabolism (97). Although these studies focus on the intestine, the “barrier damage-inflammation-abnormal energy metabolism” pathway is likely conserved in the oral-systemic axis. In patients with diabetes mellitus, periodontitis can exacerbate insulin resistance and hyperglycemia, and translocate periodontal pathogens and Th17 cells into the gut to aggravate intestinal inflammation, resulting in a vicious cycle (95). Furthermore, similar to the intestine, the integrity of the oral barrier may regulate insulin sensitivity by modulating systemic inflammatory cytokine levels. For example, supplementation with specific probiotics can ameliorate sirolimus-induced gut microbial dysbiosis and alleviate the associated systemic inflammation, dyslipidemia and insulin resistance (97). This also suggests that repairing mucosal barriers such as the oral cavity and intestine may be a novel strategy to break the vicious cycle of diabetes and its complications.

The current literature supports several distinct interpretations of the oral-systemic axis rather than a single uniform causal model. First, mechanistic and animal studies provide comparatively strong evidence that systemic dysmetabolism, especially hyperglycemia, acts upstream to impair the oral epithelial, salivary, and innate immune barrier functions; in this context, oral barrier dysfunction is best understood primarily as a consequence of systemic disease (93, 94). Second, clinical and translational observations suggest that oral barrier injury, periodontitis-associated dysbiosis, and inflammatory spillover may amplify metabolic inflammation and worsen glycemic control, thereby contributing to bidirectional reinforcement; however, these data do not establish direct oral mucosal causation (95, 96). Third, oral barrier abnormalities may also have value as predictive or monitoring markers of systemic disease burden, although this role remains provisional because most human studies are cross-sectional and heterogeneous, and vulnerable to confounding by oral hygiene, smoking, medication exposure, dietary patterns, and coexisting periodontal pathology (95, 96). Altogether, oral barrier dysfunction may serve as a downstream consequence, a disease modifier, or a potential biomarker depending on context and study design, and definitive causal inference will require longitudinal cohorts, barrier-specific biomarkers, and direct evidence that restoration of oral barrier integrity independently improves systemic metabolic endpoints (97).

### 5.2 Mucositis and barrier collapse induced by chemoradiotherapy

**Figure 8:**
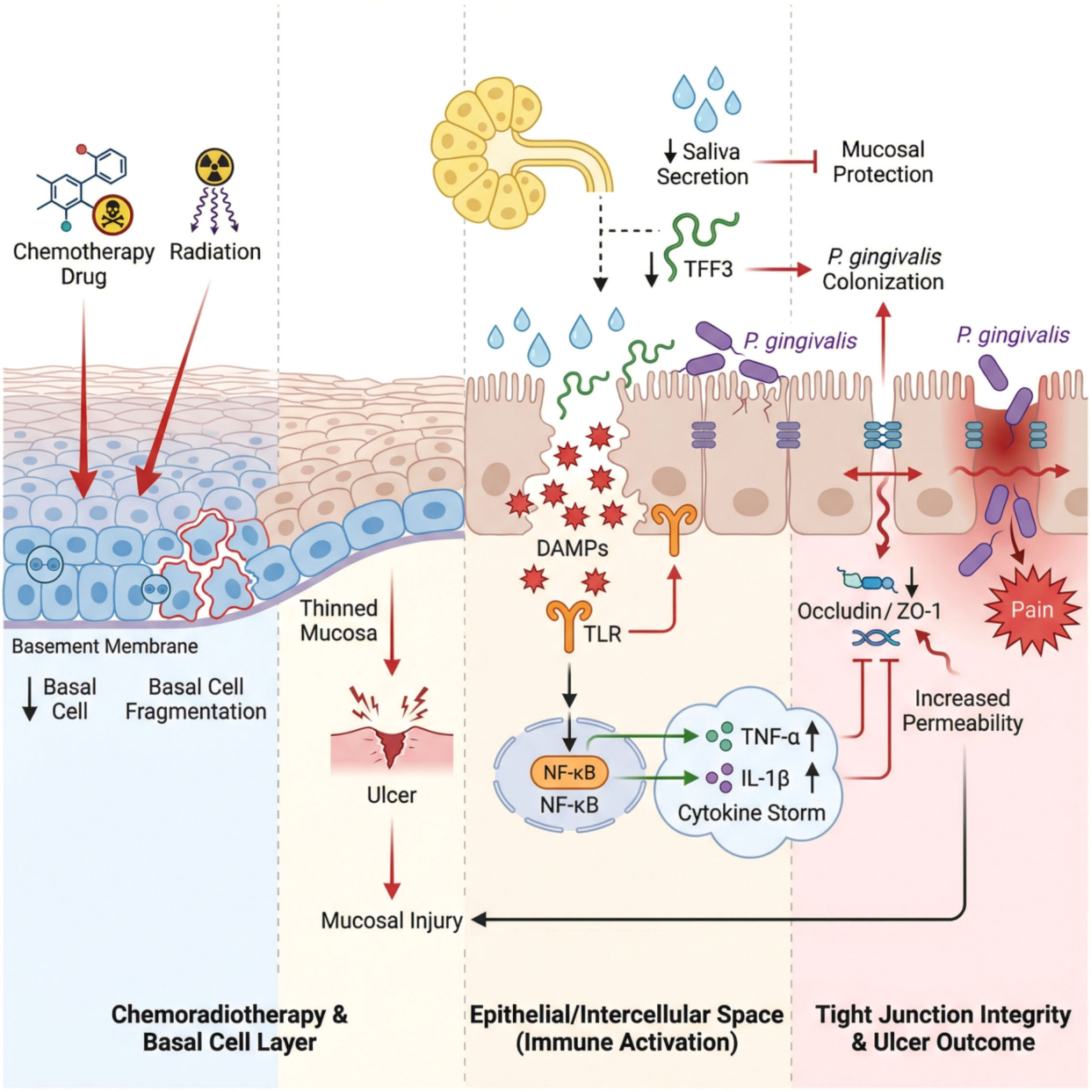
Pathological cascade of oral mucosal injury mediated by chemoradiotherapy.

Chemotherapy and radiotherapy are major causes of oral mucositis and barrier function disruption (98) (**Figure 8**). Both chemotherapeutic drugs and ionizing radiation primarily target rapidly proliferating cells, such as the basal cells in the oral epidermis that play a critical role in tissue repair and maintaining epidermal integrity. Extensive damage to the basal cells during chemotherapy and radiotherapy impairs epidermal repair function, leading to mucosal thinning and even atrophy, which can progress to painful ulcers and complete disruption of the physical barrier. This direct cytotoxic effect is the first stage of mucosal barrier damage and can trigger secondary injury in the second stage.

In addition to physical barrier disruption, post-therapy microbial dysbiosis and salivary gland injury cause concurrent impairment of the microbial and chemical barriers (99). Reduced saliva secretion diminishes its flushing, moisturizing and protective effects on the oral mucosa, and also decreases the levels of several proteins with bactericidal and mucosal repair properties, including trefoil factors (TFFs). The TFF3 peptide is an important salivary protective factor that plays a role in wound healing and antibacterial activity. Deficiency of TFF3 disrupts the oral microenvironment, and creates conditions for the colonization and invasion of opportunistic pathogens like *P. gingivalis* (98). The loss of the microbial barrier, and the impaired chemical barrier function of saliva can lead to severe secondary infections, thereby exacerbating pain and systemic inflammatory burden.

The DAMPs released from directly damaged cells activate a robust innate immune response, including the initiation of PRRs such as TLRs and downstream signaling pathways such as NF-κB (100), which increase production of inflammatory cytokines such as TNF-α and IL-1β, resulting in a “cytokine storm”. Studies show that TNF-α can directly exacerbate tissue damage in the inflammatory microenvironment and indirectly inhibit the epithelial healing process through the TLR2-β-catenin pathway (98). Excessive and persistent inflammatory stress not only fails to eliminate risk factors but also impairs the expression of tight junction-related proteins (occludin and ZO-1) (101), which increases epithelial permeability and inhibits epithelial cell regeneration, creating a vicious cycle of damage and impaired repair that significantly delays mucosal healing (100).

## 6 Microbiota-targeted intervention: remodeling the ecological barrier

### 6.1 Application of probiotics, prebiotics and postbiotics

**Figure 9:**
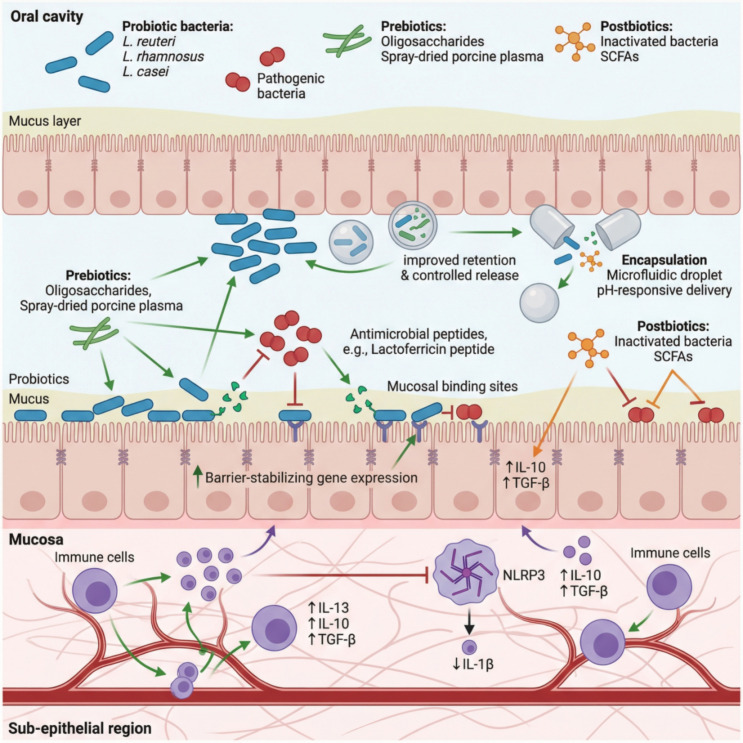
Oral microecological regulation mechanisms of probiotics, prebiotics and postbiotics.

Certain probiotics contribute to the oral mucosal barrier repair through multiple mechanisms (102) (**Figure 9**). For example, *Limosilactobacillus reuteri* regulates the intestinal mucosal barrier function by producing AMPs such as lactoferrin and lactoferricin, which inhibit pathogens and modulate host immune responses (103). Supplementation with *Lactobacillus rhamnosus* has been shown to alleviate the severity of *Toxoplasma gondii* infection in the acute phase by increasing the recruitment of immune cells to the intestinal mucosa (104) and reducing larval invasion through competitive exclusion. This protective effect appears to be associated with local immunoregulation; although the specific mechanism may not depend on IL-4, IL-10 or IFN-γ, the upregulation of IL-13 during early infection may be involved (104). In addition, probiotics such as *Lactobacillus casei* ATCC 393 and its fermented products can mitigate barrier damage by reducing the secretion of IL-1β via inactivation of the NLRP3 inflammasome pathway, and increasing levels of the anti-inflammatory cytokine IL-10 (105). Taken together, probiotics can improve intestinal mucosal barrier function through competitive exclusion of pathogenic bacteria, synthesis of bacteriocins, and activation of the anti-inflammatory program in intestinal epithelial cells (102, 106).

Prebiotics and postbiotics are also being considered as oral barrier-targeted adjuncts as they may reproduce some ecological or immunologic benefits of live probiotics, while improving safety and formulation stability (107–109). They offer advantages like prolonged storage and standardized composition, and can be used in immunocompromised patients. Potential mechanisms include selective support of health-associated oral communities, suppression of pathobiont expansion, delivery of SCFAs or other immunoactive metabolites, and modulation of epithelial and myeloid inflammatory tone (108, 109). However, there is limited evidence in the context of oral barrier function. Many prebiotic and postbiotic mechanisms – including oligosaccharide-driven microbial enrichment and butyrate-centered barrier repair – have been established in gut disease models (106–108) and should therefore be regarded as translational rationale rather than direct proof for oral mucosal disorders. Further oral-specific studies are needed to determine which compounds, doses and delivery platforms are most suitable for treating gingival inflammation, recurrent ulceration, oral mucositis and other distinct barrier phenotypes.

Conventional oral probiotic preparations must pass through the harsh gastrointestinal environment to reach the gut, and thus cannot exert a direct therapeutic effect on local oral diseases. Therefore, it is necessary to develop local drug delivery systems for oral probiotics to increase their colonization rate and prolong residence in the oral cavity, and enable sustained release. Microencapsulation technology is an effective strategy: for example, microencapsulation of *L. reuteri* engineered to express AMPs can protect the bacteria from gastric acid damage, and achieve targeted delivery to the gut for modulation of local immunity (103). Although this study focuses on the intestinal mucosa, the same principle can be applied to the design of oral delivery systems, such as sustained-release lozenges, mouthwashes or gels that can prolong the contact between probiotics or their postbiotic components and the oral mucosa. For example, pH-responsive biomineralization can be used to prepare a probiotic delivery system that forms a self-replicating “antigen factory” in the gut (mimicking the oral microenvironment), and exhibits mucosal retention and resistance to gastric acids. Microfluidic-based encapsulation strategy has been used to fabricate microparticles containing probiotics (*Escherichia coli* Nissle 1917) and prebiotics (sodium alginate and inulin gel), which significantly improve their stability and retention time in the gastrointestinal environment. Likewise, delivery systems can be designed that improve the efficacy of probiotics, prebiotics and postbiotics against oral mucosal diseases by protecting the active substances, enhancing mucoadhesion and enabling controlled release.

### 6.2 Precision microbiota transplantation and phage therapy

**Figure 10:**
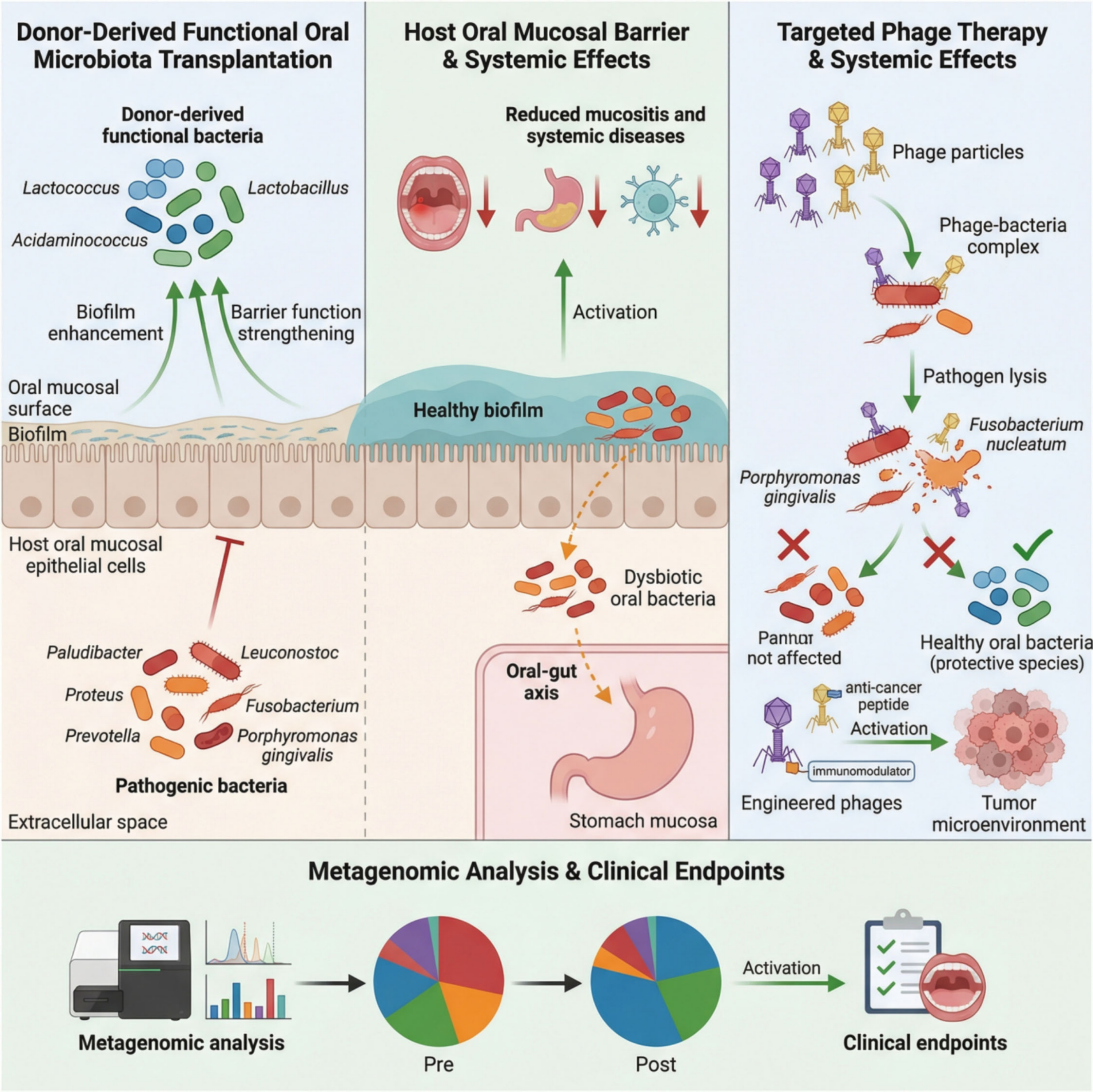
Donor-derived functional oral microbiota transplantation and targeted phage therapy.

Specific transplantation of donor-derived functional microbiota can effectively reconstruct the host’s oral mucosal barrier system. Oral microbial dysbiosis is associated with the development and progression of many diseases; for example, changes in the oral microbial composition of patients undergoing hematopoietic stem cell transplantation (HSCT) has been directly correlated with the severity of oral mucositis (109). Studies show that a higher relative abundance of *Paludibacter*, *Leuconostoc* and *Proteus* in the saliva is associated with more severe oral mucositis, while *Lactococcus* and *Acidaminococcus* exert a protective effect (109). Similarly, in pediatric patients undergoing allogeneic HSCT, oral mucositis is associated with an increase in *Fusobacterium* and *Prevotella*. This suggests that transplantation of a healthy oral microbiota rich in protective bacteria (*Lactobacillus*) may help inhibit cariogenic or periodontal pathogenic bacteria, correct dysbiosis, and improve clinical outcomes. Oral microbial dysbiosis can also influence the development of systemic diseases through the “oral-gut axis”. For example, in chronic atrophic gastritis, oral-derived *P. gingivalis* translocates into the stomach and exacerbates gastric mucosal inflammation and precancerous lesions. Thus, reconstructing the oral microecology via transplantation of a healthy oral microbiota can not only treat local infections but may also alleviate systemic autoimmune and metabolic diseases (**Figure 10**). However, the clinical translation of this approach faces significant challenges, such as the standardization of microbiota preparations, and concerns related to long-term safety and efficacy.

Bacteriophages can selectively kill pathogenic bacteria without affecting the normal oral microbiota, and offer a suitable alternative for addressing oral infections caused by antibiotic resistance. Since the oral cavity contains multispecies biofilms on both mucosal and hard-tissue surfaces, phages are especially attractive for selectively depleting pathogens while sparing beneficial commensals. Candidate oral targets include *P. gingivalis* and *F. nucleatum*, which are linked to periodontal inflammation, barrier destabilization, in the case of *F. nucleatum*, pro-tumorigenic immune remodeling in OSCC (9, 67). Engineered phages may also provide a platform for targeted delivery of antimicrobial or immunomodulatory cargo within oral biofilms or tumor-associated microbial niches. Nevertheless, oral phage therapy remains at the preclinical stage, and its feasibility will depend on resolving issues pertaining to the oral environment, such as penetration of dense biofilms, persistence under salivary flow, narrow host range, bacterial resistance, and possible neutralization by host immunity.

Precision microbiota transplantation and phage therapy therefore face shared translational challenges before they can be incorporated into oral medicine. First, therapeutic preparations need rigorous standardization, including donor or community selection, screening workflows, manufacturing pipelines, dosing schedules, and formulations such as mouthwash, gel, lozenge or site-retentive biomaterial coating. Second, long-term safety and efficacy must be evaluated in oral-specific clinical settings, particularly with respect to pathogen transfer, off-target ecological disturbance, host-range uncertainty, and immunological compatibility. Third, outcome assessment should include oral-specific microbiological, epithelial, immunological and healing readouts in addition to systemic signs, ideally integrating metagenomic profiling with clinically meaningful endpoints such as ulcer duration, mucositis grade, periodontal inflammatory burden or local recurrence risk. Although studies on gut microbiota therapeutics remain informative, oral-specific validation is necessary due to differences in engraftment rules, barrier architecture and ecological pressures between the oral cavity and the intestine.

### 6.3 Comparative clinical translation of emerging barrier-targeted strategies

From the perspective of clinical translation, the candidate interventions discussed above differ substantially in maturity, safety profile, evidence strength and implementation barriers. Among them, probiotics and related biotic approaches currently have the most direct human evidence. Recent meta-analyses suggest that these formulations can reduce the severity of treatment-related oral mucositis, and a randomized controlled trial of *Streptococcus salivarius* K12 supplementation in patients receiving radiotherapy for malignant head and neck tumors demonstrated reductions in the incidence, onset and duration of severe oral mucositis with an overall favorable safety profile (110–112). Nevertheless, their efficacy is dependent on the bacterial strain, dose, and formulation, and may be influenced by baseline microbiota composition, concurrent antibiotic exposure, host immune status and treatment setting. Furthermore, careful product selection and surveillance remain imperative in severely immunocompromised individuals. In this context, postbiotics may offer practical translational advantages such as safety, storage stability, manufacturing reproducibility and regulatory standardization, although oral disease-specific clinical data are still comparatively limited (107–109, 113).

By comparison, nanocarrier- and biomaterial-based delivery systems show significant potential due to their ability to prolong mucosal residence of the cargo, protect labile payloads, improve local retention within the highly dynamic oral environment, and enable spatiotemporally controlled drug release (114, 115). However, these platforms are currently at the preclinical or early translational stage, and their clinical progression is constrained by long-term mucosal biocompatibility, batch-to-batch reproducibility, sterilization, large-scale manufacturing, and regulatory classification. Phage therapy and precision microbiota transplantation are conceptually attractive because they either deplete pathogens selectively, or restore the ecological balance while preserving commensal communities. Nevertheless, their translation is currently limited by donor or product standardization, narrow or unstable host range, emergence of phage resistance, quality-control requirements, dosing schedules, and the lack of validated efficacy end points tailored to oral barrier disorders (113, 116–118). Overall, standardized biotic formulations appear closest to near-term clinical implementation, whereas nanocarrier systems, phage therapy and microbiota transplantation will require more rigorous safety benchmarking, mechanism-linked biomarkers, and adequately powered clinical studies before routine adoption. To facilitate comparison across intervention classes and to better reflect differences in translational maturity, we have summarized the major barrier-targeted strategies in **Table 1** according to therapeutic target, mechanism of action, evidence level, major advantages, current limitations, and possible oral indications.

**Table 1:** Translational status of targeted intervention strategies for oral mucosal barrier disorders.

Strategy	Therapeutic target	Mechanism of action	Evidence/maturity	Major advantages	Current limitations	Possible oral indications
Probiotics	Dysbiosis; weak colonization resistance; inflammatory amplification	Competitive exclusion; bacteriocins or metabolites; epithelial-immune modulation	Best oral maturity; preclinical plus early clinical or RCT data, especially in mucositis	Familiar, non-invasive, multi-target	Strain and formulation dependence; colonization variability; caution in severe immunosuppression	Oral mucositis; dysbiosis-linked inflammatory lesions; adjunct in periodontal or ulcerative disease
Postbiotics	Inflammatory signaling; barrier-repair defects; microbial imbalance without live organisms	Defined inactivated microbes or bioactive products such as SCFAs to modulate immunity and repair	Promising preclinical evidence; oral clinical data still limited	Better stability, reproducibility, and safety than live biotics	Mechanistic heterogeneity; few oral trials; unclear dose-response and regulatory category	Mucositis; oral inflammatory lesions; barrier-repair settings where live probiotics are unsuitable
Precision microbiota-targeted therapies	Disease-specific ecological disruption; loss of beneficial consortia; pathobiont enrichment	Defined consortia, tailored transplantation, or ecological editing to restore microbial balance	Early oral translation; rationale strong, standardization limited	Potentially personalized ecological correction	Safety, engraftment durability, product standardization, and endpoints remain unresolved	Refractory dysbiosis-associated disease; recurrent mucositis; selected periodontal or premalignant settings
Phage-based approaches	Specific pathogenic bacteria in oral biofilms, including resistant or tumor-associated species	Selective bacteriolysis; possible engineered delivery of immunomodulatory or anticancer payloads	Strong preclinical rationale; oral clinical translation still early	High specificity; microbiota-sparing potential	Narrow host range; phage resistance; biofilm penetration and manufacturing challenges	Periodontal pathogen targeting; biofilm-associated infection; microbiota-linked oral cancer adjuncts
Nanodelivery or biomaterial-based systems	Poor local drug retention; fragile cargo; lesions requiring controlled release	Mucoadhesive or stimulus-responsive delivery of drugs, nucleic acids, biologics, or microbial products	Robust preclinical evidence; limited routine clinical adoption	Enhanced residence, cargo protection, site specificity, and spatiotemporal control	Biocompatibility, sterilization, scale-up, reproducibility, and regulatory classification remain barriers	Oral ulcers; oral mucositis; localized inflammatory lesions; precision delivery to premalignant or tumor-related niches

## 7 Future perspectives

Future advances in this field will likely depend on the convergence of high-resolution biology, computational analytics, and precision intervention platforms. Artificial intelligence-assisted microbiome analysis may enable a transition from descriptive profiling toward clinically actionable decoding of oral ecological states by integrating taxonomic, metatranscriptomic, metabolomic, spatial, and host immune data. In this framework, machine-learning models may help identify functionally relevant microbial signatures, predict disease trajectories, and stratify patients according to the likelihood of response to barrier-directed or microbiota-targeted therapy (119). At the same time, rigorous model development, standardized sampling, transparent reporting, and external validation across populations will be essential to avoid overfitting and to ensure clinical interpretability (119).

A second major direction is the evolution of microbiota-targeted intervention from empirical supplementation toward precision microbiota therapy. Rather than relying solely on broadly defined probiotics, future strategies are likely to incorporate defined microbial consortia, rationally selected postbiotics, bacteriophage-guided ecological editing, and engineered commensals designed to restore barrier-supportive functions in a context-specific manner (120). In the oral cavity, such approaches will need to account for site-specific ecological variation, salivary flow, biofilm architecture, and host inflammatory tone, while also incorporating delivery systems capable of improving local retention and spatiotemporal control (114, 120).

Personalized mucosal immunomodulation is also a promising translational direction. Recent evidence indicates that oral mucosal immunity is spatially organized across distinct epithelial niches, suggesting that future interventions may need to be tailored not only to disease category but also to anatomical subsite and local immune architecture (121). In parallel, advanced oral mucosal delivery platforms, including mucoadhesive systems and microneedle-based devices, offer opportunities for site-specific administration of vaccines, biologics, nucleic acids, and other immunoregulatory agents (114, 122). Such technologies may help increase local efficacy while minimizing systemic exposure and off-target immune activation.

A particularly actionable next step will be the establishment of a molecular classification framework for oral mucosal barrier dysfunction. Rather than grouping heterogeneous disorders under a single concept of “barrier injury,” future studies should define reproducible molecular subtypes according to epithelial state, immune polarization, microbial ecology, stromal activation, and repair capacity (8). Such a framework could distinguish, for example, ulcerative-inflammatory, dysbiosis-dominant, fibrotic-remodeling, therapy-injured, and premalignant barrier states, thereby enabling more rational patient stratification, mechanism-based trial design, and matching of therapeutic targets to disease context. Furthermore, it is also crucial to develop saliva- or mucosa-based biomarker systems that are practical for longitudinal monitoring and clinical decision support. Candidate biomarkers may include cytokine or alarmin panels, AMPs, junction-associated proteins, epithelial stress markers, extracellular vesicle cargo, microbiota-derived signatures, and spatially informed immune-cell states. To become clinically actionable however, these markers will need standardized sampling procedures, clear analytical thresholds, external validation across oral subsites and disease stages, and ability to add predictive value beyond conventional clinical assessment (123).

Mechanistic translation will also depend on experimental platforms that more faithfully recapitulate the oral barrier niche. Oral organoid-microbiota-immune co-culture systems represent a particularly important frontier because they may permit causal testing of epithelial-microbial-immune interactions under controlled conditions. When combined with stromal components, salivary factors, or microfluidic perfusion, such models could help dissect how barrier disruption gradients emerge, why some lesions progress toward chronicity or dysplasia, and which interventions restore durable barrier competence rather than only transient anti-inflammatory effects. Greater emphasis should be placed on precision local delivery systems that can translate mechanistic insights into site-specific therapy. Future platforms should move beyond simple topical exposure and instead optimize mucosal residence time, penetration depth, release kinetics, lesion selectivity, and compatibility with biologics, nucleic acids, postbiotics, or microbiota-modulating agents. The most impactful translational pathway will likely link molecular classification, biomarker-guided patient selection, and smart local delivery into an integrated framework for personalized barrier-restorative therapy in disorders such as oral mucositis, oral lichen planus, recurrent ulceration, and premalignant inflammatory lesions (124).

Finally, patient-derived oral organoids and related ex vivo models may provide an important bridge between mechanism and individualized therapy. By preserving key genetic, phenotypic, and microenvironmental features of patient tissues, these platforms may support drug screening, immune-response testing, and the rational selection of personalized barrier-restorative regimens for disorders such as oral squamous cell carcinoma, oral lichen planus, and oral mucositis (124). Taken together, the future of oral mucosal barrier research lies in integrating multi-omics, artificial intelligence, functional microbiology, precision delivery, and patient-specific modeling to move from generalized barrier support toward predictive, mechanism-guided, and individualized intervention.

## 8 Conclusion

The oral mucosa is the body’s first gateway against exogenous invasion, and an intact mucosal barrier is of great significance for both local and systemic health. This review elaborates on the complex, multilevel and dynamically balanced oral mucosal immune barrier that is composed of physical, chemical, microbial and immune cellular barriers, and is precisely regulated by the intricate crosstalk among epithelial cells, immune cells and colonizing microorganisms. From a research perspective, the field has evolved from the description of static structures to an understanding of dynamic remodeling. In the context of this review, barrier remodeling denotes the adaptive or maladaptive reconfiguration of the barrier system, whereas barrier disruption and barrier dysfunction respectively refer to acute structural breach and sustained loss of barrier competence. In systemic diseases however, oral barrier dysfunction may represent a downstream consequence of systemic pathology, a mediator of bidirectional inflammation, or a predictive correlate of disease burden, whereas direct causality remains incompletely established in most clinical settings. This also suggests that the evaluation and intervention of the oral mucosal barrier will serve as a bridge connecting oral and systemic health.

In recent years, the application of advanced assessment technologies such as single-cell sequencing and spatial transcriptomics has enabled the spatiotemporal characterization of cellular heterogeneity, molecular pathways and intercellular interactions during barrier remodeling, and identified distinct cell types and molecular targets. At a more mechanistic level, these technologies can pinpoint rare pathogenic epithelial states, resolve immune-spatial communication networks, track transition gradients from homeostatic to disrupted niches, and prioritize biomarkers or therapeutic targets on the basis of cell-state specificity rather than bulk-tissue averages. This provides a solid theoretical basis for the shift from non-specific supportive therapy to targeted precision intervention; however, inconsistencies exist among different studies regarding the functions of specific cell subsets, the regulatory status of individual signaling pathways, or the impact of specific microbial changes. Resolving these inconsistencies requires an understanding of the functional redundancy and context specificity of the barrier system: the major injury and repair mechanisms may vary across different diseases or different stages of the same disease. Thus, future mechanistic research should combine more detailed clinical staging and molecular subtyping to identify the key drivers under specific conditions.

A greater understanding of the mechanisms underlying oral barrier dysfunction opens the possibility of personalization and targeted therapeutic interventions, including microecological regulation at the source, targeted immunomodulation of the host, and the use of novel drug delivery forms to achieve site-specific high-concentration release and mechanical barrier effects. These measures are not mutually exclusive but complementary, aiming to reconstruct the dynamic balance of the barrier system at different levels.

Looking ahead, the research hotspots and challenges in this field lie in integration and translation. On the one hand, it is necessary to further integrate the findings of high-throughput omics with clinical phenotypes and prognosis, and establish a classification of barrier function subtypes based on molecular markers to guide targeted repair strategies. On the other hand, it is essential to carry out prospective cohort observations and interventional trials with rational design to further clarify and verify the bidirectional causal relationship between oral mucosal barrier health and systemic diseases. The ultimate goal is to elevate the monitoring and maintenance of the oral mucosal barrier to an operable preventive and therapeutic strategy for promoting systemic health.
