# Correction: Remodeling mechanisms and intervention strategies of the oral mucosal immune barrier

**DOI:** 10.3389/fimmu.2026.1923888

**Published:** 2026-07-08

**Authors:** Zun Wang, Yaohua Guo, Yaguang Fan, Xuebing Li, Wang Shen

**Affiliations:** 1 Sichuan University, West China School of Stomatology, Chengdu, China; 2 West China Hospital of Sichuan University, Chengdu, China; 3 Tianjin Medical University General Hospital, Tianjin Lung Cancer Institute, Department of Lung Cancer Surgery, Tianjin Key Laboratory of Lung Cancer Metastasis and Tumor Microenvironment, Tianjin, China

**Keywords:** barrier function, epithelial barrier, immune remodeling, intervention strategies, microbiota, oral mucosal immunity

In the published article, the 3rd affiliation for authors “Yaguang Fan, Xuebing Li and Wang Shen” was erroneously stated as “Tianjin Medical University Cancer Institute & Hospital, Tianjin Lung Cancer Institute, Tianjin, China”. The correct affiliation is “Tianjin Medical University General Hospital, Tianjin Lung Cancer Institute, Department of Lung Cancer Surgery, Tianjin Key Laboratory of Lung Cancer Metastasis and Tumor Microenvironment, Tianjin, China”.

The original version of this article has been updated.

