# Correction: Remodeling mechanisms and intervention strategies of the oral mucosal immune barrier

**DOI:** 10.3389/fimmu.2026.1935862

**Published:** 2026-07-30

**Authors:** Zun Wang, Yaohua Guo, Yaguang Fan, Xuebing Li, Wang Shen

**Affiliations:** 1 Sichuan University, West China School of Stomatology, Chengdu, China; 2 West China Hospital of Sichuan University, Chengdu, China; 3 Tianjin Medical University General Hospital, Tianjin Lung Cancer Institute, Department of Lung Cancer Surgery, Tianjin Key Laboratory of Lung Cancer Metastasis and Tumor Microenvironment, Tianjin, China

**Keywords:** barrier function, epithelial barrier, immune remodeling, intervention strategies, microbiota, oral mucosal immunity

In the published article, the equal first-author annotation for “Zun Wang and Yaohua Guo” was absent in the author section. The following sentence has been added to the author information section:

“^†^These authors have contributed equally to this work and share first authorship”

The original version of this article has been updated.

